# Newborn Screening by DNA-First: Systematic Evaluation of the Eligibility of Inherited Metabolic Disorders Based on Treatability

**DOI:** 10.3390/ijns11010001

**Published:** 2024-12-28

**Authors:** Abigail Veldman, Birgit Sikkema-Raddatz, Terry G. J. Derks, Clara D. M. van Karnebeek, M. B. Gea Kiewiet, Margaretha F. Mulder, Marcel R. Nelen, M. Estela Rubio-Gozalbo, Richard J. Sinke, Monique G. de Sain-van der Velden, Gepke Visser, Maaike C. de Vries, Dineke Westra, Monique Williams, Ron A. Wevers, M. Rebecca Heiner-Fokkema, Francjan J. van Spronsen

**Affiliations:** 1 Division of Metabolic Diseases, Beatrix Children’s Hospital, University Medical Center Groningen, University of Groningen, 9718 GZ Groningen, The Netherlands; 2 Department of Genetics, University Medical Center Groningen, University of Groningen, 9713 GZ Groningen, The Netherlands; 3 Departments of Pediatrics and Human Genetics, Emma Center for Personalized Medicine, Amsterdam Gastroenterology Endocrinology Metabolism, Amsterdam University Medical Center, 1105 AZ Amsterdam, The Netherlands; 4 Department of Human Genetics, Radboud University Medical Center, 6500 HB Nijmegen, The Netherlands; 5 Division of Metabolic Diseases, Department of Pediatrics, Maastricht University Medical Center, 6229 HX Maastricht, The Netherlands; 6 Department of Genetics, Section Metabolic Diagnostics, University Medical Center Utrecht, 3584 CX Utrecht, The Netherlands; 7 Division of Metabolic Disorders, Department of Pediatrics, Radboud University Medical Center, 6525 GA Nijmegen, The Netherlands; 8 Center for Lysosomal and Metabolic Diseases, Department of Pediatrics, Erasmus University Medical Center, 3015 GD Rotterdam, The Netherlands; 9 Laboratory of Metabolic Diseases, Department of Laboratory Medicine, University Medical Center Groningen, University of Groningen, 9718 GZ Groningen, The Netherlands

**Keywords:** inherited metabolic disease, inborn errors of metabolism, newborn screening, treatability, heel prick, Wilson and Jungner criteria, next-generation sequencing, genetics-first

## Abstract

The biomarker-based Dutch Newborn Screening (NBS) panel (as of 2024) comprises 19 inherited metabolic disorders (IMDs). With the use of next-generation sequencing (NGS) as a first-tier screen, NBS could expand to include IMDs that lack a reliable biochemical footprint in dried blood spots, while also reducing secondary findings. To be eligible for inclusion in NBS, an IMD needs to fulfill the Wilson and Jungner criteria, with treatability being one of the most important criteria. In this study, we aimed to identify IMDs eligible for DNA-first NBS when considering only treatability in the context of NBS as a prerequisite. First, three independent reviewers performed a systematic literature review of the 1459 genotypic IMDs and their causative gene(s), as described in the International Classification of Inherited Metabolic Disorders (dated 1 February 2021), applying 16 criteria to exclude non-treatable disorders. Eligible disorders were then discussed in three online meetings with a project group of clinical laboratory geneticists, medical laboratory specialists specialized in IMD, and pediatricians with expertise in IMDs. Based on treatability, we identified 100 genes, causing 95 IMDs, as eligible for NBS, including 42 causal genes for the IMDs in the current biomarker-based NBS. The other 58 genes are primarily associated with treatable defects in amino acid metabolism and fatty acid oxidation. Other IMDs were excluded, most often because of insufficient literature. As the evaluation of treatability was not straightforward, we recommend the development of standardized treatability scores for the inclusion of IMDs in NBS.

## 1. Introduction

Newborn screening (NBS) is an important and successful national public health program in the Netherlands [1,2,3,4]. Its aim is early, preferably pre-symptomatic, detection of disorders in which timely intervention can reduce morbidity and mortality. The Dutch NBS panel (as of 2024) consists of 27 disorders, including 19 monogenetic inherited metabolic disorders (IMDs) [4,5,6]. Almost all NBS tests for IMDs are based on biochemical tests that measure either a metabolite (or a combination of metabolites) or enzyme activities. These screening methods are sensitive, but they can have low positive predictive values (PPVs) [1,2,3,4]. This is particularly the case for IMDs screened for using metabolite concentrations, where abnormal concentrations may also identify non-targeted disorders, resulting in PPVs that vary between 8% and 92% [4]. For example, screening for phenylalanine hydroxylase deficiency (phenylketonuria (PKU)) (*PAH*, MIM *612349, #261600) is performed by screening for an elevated phenylalanine concentration in a dried blood spot (DBS). However, high phenylalanine levels will also detect DNAJC12 deficiency (*DNAJC12*, MIM *606060, #617384); four defects in the metabolism of the cofactor tetrahydrobiopterin due to bi-allelic variants in *PTS* (MIM *612719, #261640), *PCBD1* (MIM *126090, #264070), *QDPR* (MIM *612676, #261630), and *GCH1* (MIM *600225, #128230, #233910) (only autosomal recessive type) [7]; and children with liver disease. Due to the low prevalence of these other defects, the PPV for PKU was 92% from 2018 to 2022 [4]. Screening for tyrosinemia type I (*FAH*, MIM *613871, #276700) using DBS succinylacetone as a biomarker had a PPV of 9% from 2018 to 2022 [4,8], this biomarker often reveals variants in *GSTZ1* (MIM *603758, #617596), which causes the non-clinical entity maleylacetoacetate isomerase deficiency [9].

A low PPV for some IMDs can also be partially explained by IMDs or enzyme deficiencies in the newborn’s mother that lead to false-positive NBS hits [10]. For example, abnormal concentrations of the biomarker methylmalonic acid may derive from an IMD such as methylmalonic acidemia (MMA); however, increased concentrations of the biomarker methylmalonic acid can be caused by more than 20 genes (including *MMUT*, MIM *609058, #251000; *MMAA*, MIM *607481, #251100; *MMAB*, MIM *607568, #251110; *MMADHC*, MIM *609831, #277410; *MMACHC*, MIM *611935, #277400; or *MCEE*, MIM *608419 #251120), or from a maternal vitamin B12 deficiency that is often nutritional in origin [7]. In addition, maternal vitamin B2 deficiency could lead to acylcarnitine and organic acid profiles like those observed in multiple acyl-CoA dehydrogenase deficiency (MIM #231680; *ETFA*, *608053; *ETFB* *130410; *ETFDH*, *231675) or very long chain acyl-CoA dehydrogenase (*ACADVL*, MIM *609575 #201475) [10]. False-positive screening results due to maternal IMDs are reported for PKU (*PAH*, MIM *612349, #261600), 3-methylcrotonylglycinuria (*MCCC1*, MIM *609010, #210200; *MCCC2*, MIM *609014, #210210), primary carnitine deficiency (*SLC22A5*, MIM *603377, #212140), medium-chain acyl-CoA dehydrogenase deficiency (MCAD) (*ACADM*, MIM *607008, #201450), and glutaric acidemia type 1 (*GDCH*, MIM *606601, #231670) [7,11]. An overview of all IMDs in the current Dutch NBS and possible secondary findings is presented in Table 1.

In recent years, next-generation sequencing (NGS) has been investigated as a second-tier approach to conventional NBS [14,15,16,17,18,19,20,21,22,23,24], and some studies have reported the advantages of using NGS as a first-tier test compared to the biochemistry-first approach [17,23,25,26,27]. An NGS-first-based NBS would theoretically allow screening for all disorders with a (mono)genetic background, including those without clear biochemical footprints [28,29,30]. Without the false-positives due to non-specific findings, NGS could increase the PPV of NBS. Given these technical possibilities, the Wilson and Jungner (W&J) criteria and their revised versions by Anderman et al. [31,32,33] will become increasingly important for the selection of disorders to be included in NBS [34,35,36]. These criteria form the basis of safe and ethically acceptable implementation of new disorders into public health screening programs. One of the key W&J criteria is: ‘*There should be an accepted treatment for patients with recognized disease*’ [32]. In the realm of genetic screening, Andermann et al. [31] proposed a framework to guide policymaking in this area. They outlined twenty criteria, including criterion 17, which focuses on intervention: ‘*There should be an accepted intervention (ex. prevention, treatment, family planning) that forms part of a coherent management system*’ [31]. These criteria form a groundwork for selecting treatable disorders for NBS. However, in this context, there is a difference between the availability and effectiveness of treatment and the benefit of early treatment or intervention [34]. Moreover, a clear definition and boundary between ‘actionable’, ‘treatable’, and ‘curable’ disorders are still being debated [35,36].

As early treatability is considered a prerequisite and one of the most important criteria [34,37,38] for NBS, we aimed to develop a list of disorders eligible for NBS. This list is primarily based on a review of the literature to assess treatability, followed by defining exclusion criteria and expert meetings to come to a consensus. We also share and discuss the approach we chose to identify the most important challenges in defining treatability and related criteria. We believe this discussion will be helpful to others engaged in the worldwide effort to further develop criteria to include and exclude disorders in NBS, where transparency is of utmost importance.

## 2. Materials and Methods

### 2.1. Project Team Participants

The core team invited members of the Dutch Advisory Committee Newborn Screening for IMDs (ANS-IMD) and researchers in the Dutch NGS-first for NBS (NGSf4NBS) project [17] to participate in the project team. The project team consisted of metabolic pediatricians (*N* = 7), medical laboratory specialists specialized in IMDs (*N* = 2), and clinical laboratory geneticists (*N* = 4). All Dutch University Medical Centers involved in the care of patients with IMDs and the National Institute for Public Health and Environment (RIVM) were represented in the project team. All members were offered, and accepted, authorship by participating. The core team was: A.V. (medical student), M.R.H-F (medical laboratory specialists specialized in IMDs), and F.J.v.S. (metabolic pediatrician). The study was carried out between October 2020 and June 2021, a period during which the COVID-19 pandemic prevented live meetings. In parallel, a Delphi study was initiated to elaborate on the definition of treatability in the context of NBS [39].

### 2.2. Study Design

**Figure 1:**
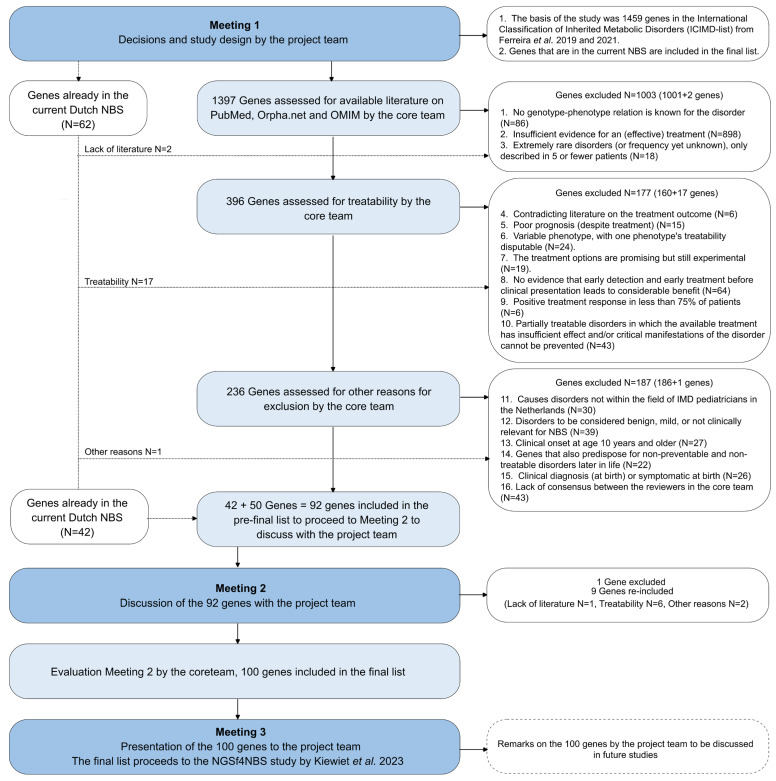
A discussion-based literature search to select genes associated with IMDs for next-generation sequencing as a first-tier approach in newborn screening. NBS = newborn screening, dark blue = project team + core team, light blue = core team only. References: [26] Kiewiet, G.; Westra, D.; de Boer, E.N.; van Berkel, E.; Hofste, T.G.; van Zweeden, M.; Derks, R.C.; Leijsten, N.F.; Ruiter-kamp-Versteeg, M.H.; Charbon, B.; et al. Future of Dutch NGS-Based Newborn Screening: Exploring the Technical Possibilities and Assessment of a Variant Classification Strategy. Int. J. Neonatal Screen. 2024, 10, 20; [48] Ferreira, C.R.; Rahman, S.; Keller, M.; Zschocke, J.; ICIMD Advisory Group. An international classification of inherited metabolic disorders (ICIMD). J. Inherit. Metab. Dis. 2021, 44, 164. [49] Ferreira, C.R.; Van Karnebeek, C.D.M.; Vockley, J.; Blau, N. A proposed nosology of inborn errors of metabolism. Genet. Med. 2019, 21, 102–106.

The study consisted of various phases, as depicted in Figure 1 (selection process Section 2.2.1, literature review by core team Section 2.2.2, and evaluation of literature review by project team Section 2.2.3). In total, three (online) meetings with the project team were arranged by members of the core team. Consensus on important decisions about the study design and the inclusion or exclusion of genes in the list of genes eligible for a genetic NBS was defined as 75% agreement in the core and project teams.

#### 2.2.1. Meeting 1: Defining Treatability and Strategy of the Selection Process

First, the core team initiated a meeting with the project team to discuss the study design and explore whether every member could agree on starting the selection procedure with the “treatability” criterion from W&J and Andermann et al. [31,32,33], rather than using a quantitative scoring matrix of all the criteria together as given by the Recommended Universal Screening Panel by the American College of Medical Genetics and Genomics (ACMG) [40,41] or other then-current international attempts to select disorders [22,42,43,44,45]. In meeting 1, we agreed to consider an IMD “treatable” if early intervention substantially improves health outcomes, consistent with the Dutch Health Council statement: “The primary outcome of NBS should be a significant benefit in health because of early intervention in disorders with a well-known natural course” [34]. At that time, the literature on “treatability” in the context of NBS was limited [42,46,47]. The project team further agreed that the IMDs selected should be only those in which the benefits of inclusion in NBS outweigh the disadvantages beyond a reasonable doubt, in line with the result of the Delphi study on treatability [39]. The project team was allowed to propose suggestions for treatability-related criteria for our literature review.

In meeting 1, it was also decided to start with the 1459 genes associated with IMDs according to the International Classification of IMD (ICIMD), as described by Ferreira et al. [48,49] and reported in the IEM-Base (accessed 1-February-2021). In addition, it was decided that IMDs included in the Dutch biomarker-based NBS at that time would be included in the group of disorders accepted for further research into NGS-first screening [4,13]. That meant that we selected all the genes associated with these IMDs, including genes unintentionally screened for since the introduction of PKU in 1974 (i.e., those found as secondary findings) [Table 1].

#### 2.2.2. Literature Review by the Core Team

After meeting 1, based on project team suggestions and the then-current literature on treatability, the core team formulated a set of exclusion criteria [Figure 1 and Appendix B] for their literature research to assess the eligibility of IMDs for NBS. The members of the core team started an independent selection process of systematically reviewing each gene to assess its eligibility for an NGS-based NBS.

Following the guidance of the exclusion criteria [Figure 1 and Appendix B], the core team searched the databases of PubMed, ORPHA, Online Mendelian Inheritance in Man (OMIM), and Google Scholar for treatment options for these IMDs and their associated gene(s). The search terms used were: the IMD name, its abbreviations or alternative names, the name of the gene(s), and the terms “treatment”, “treatability”, “improved outcome”, “intervention”, “newborn screening”, and “neonatal screening”. Reason(s) for excluding a gene were reported in a database (Microsoft Office 2021 Excel: Version 2103 (Build 13901.20312)) shared within the project team. To complete the database and consistently apply the exclusion criteria [Figure 1 and Appendix B], the core team decided to review the genes from the current NBS with the same strategy.

#### 2.2.3. Meetings 2 and 3: Evaluation of the Literature Review by the Core and Project Teams

In meeting 2, consensus was reached by members of the project group on the criteria formulated by the core team [Appendix B]. An initial list of potentially eligible genes, along with all the core team’s considerations, was presented to the project team in that meeting. The list was discussed, aiming to elicit suggestions (shared via email afterward). After meeting 2, alterations were made to the initial list. The core team members then created a final list of genes eligible for an NGS-first-based NBS. This final list was presented to the project team in meeting 3. Figure 1 shows an overview of this literature-review-based discussion, which is further elaborated in the Results.

## 3. Results

### 3.1. Genes Already Screened for in Current NBS

There are 62 genes associated with the IMDs screened for in current NBS [Table 1]. The project team, however, agreed that the phenotypes related to (likely) pathogenic variants in 20 specific genes were not eligible for inclusion in NBS (genes in blue in Table 1), resulting in a total of 42 genes. The reasons for excluding these 20 genes are further elaborated in Appendix D.

### 3.2. Results of the Literature Review of the Remaining 1397 Genes

The ICIMD list consists of 1459 genes. The literature review started with 1397 genes, excluding the 62 genes from current NBS. If an IMD is caused by pathogenic variants in different genes, each gene was reviewed separately. The criteria in Figure 1 and Appendix B were applied to the remaining 1397 unreviewed genes from the ICIMD list. The exclusion criteria, established by the core team and agreed on by the project team at meeting 2, can be divided into three main categories: 1. Lack of literature, 2. Treatability, and 3. Other reasons for exclusion. The criteria were applied in the order listed in Figure 1 and Appendix B. This does not exclude that most IMDs could also have been excluded for other reasons. Therefore, Figure 1 and Appendix B depict the first reason for exclusion, and we provide an overview of the reasons for exclusion of all genes.

#### 3.2.1. Lack of Literature and/or Evidence

Most genes (*N* = 1001) were excluded because of insufficient literature, defined as two of fewer studies of substantial quality on improved outcomes with treatment (*N* = 896) or lack of information on the correlation between genotype and phenotype (*N* = 86). In addition, extremely rare disorders were excluded (*N* = 18), i.e., when only a few patients or a single case study were found in the literature and thus there was insufficient evidence available to determine treatability or eligibility for NBS.

#### 3.2.2. Treatability

Of the remaining 396 genes, 160 were excluded because of lack of treatability in the context of NBS. In these disorders, an early diagnosis (and an early start of treatment) through NBS did not result in further improvement of outcome in patients, compared to those who presented symptomatically in the clinic.

#### 3.2.3. Other Reasons

Of the remaining 236 genes, 186 were excluded for other reasons (see Appendix B).

### 3.3. Considerations for the Final List of Genes

Combining the 42 genes in the current NBS with the 50 genes from the literature review of the remaining 1397 genes resulted in an initial list of 92 potential eligible genes. This list, together with the considerations of the core team, was presented to the project team in meeting 2. After this meeting, based on suggestions with 75% consensus in the project team, a few further alterations were made (for reasons see Appendix C). In meeting 3, a final list of 100 genes was presented to the project team. These 100 genes [Table 2], corresponding to 95 IMDs, are mainly defects in amino acid metabolism and fatty acid oxidation, as are the 42 genes (31 IMDs) already included in current NBS. Appendix A presents a more extensive list of the 100 genes including IMD names and OMIM codes.

## 4. Discussion

The aims of this study were to develop a list of disorders eligible for NBS that is primarily based on treatability and to discuss the chosen approach to identify the most important challenges in defining “early” treatability and related criteria in the context of a genetics-based NBS. Using this approach, we aimed to take a first step towards transparency about the inclusion of IMDs in a genetic NBS based on treatability, while acknowledging all W&J and Andermann criteria [31,32,33]. Our experience in practice, however, revealed that the evidence for treatability can sometimes be difficult to judge. Additionally, even when trying to evaluate only treatability, it is challenging to leave out other disorder-specific factors such as age of onset, predisposing factors, and phenotypic variability.

### 4.1. Challenges in Defining Treatability

Apart from the discussion about the inclusion or exclusion of genes, the project faced some challenges. First, the concept of treatability led to substantial discussions within our core and project teams. The definition of treatability in IMDs based on clinical presentation, used by various authors [46,47], is not the same as treatability in the sense of population-based NBS. From the W&J and Andermann criteria [31,32,33], it is clear that a treatment for a disorder within a screening program should result in an improved prognosis through early pre-symptomatic detection followed by treatment when compared to clinical presentation of symptoms followed by treatment [34]. But to what extent such improvement must be shown is unknown and hard to define. Even within the Delphi survey that we performed to further elaborate on this, it was very difficult to achieve a more solid definition [39]; however, most of the participating Dutch professionals agreed that 75% of the patients need to show a meaningful improvement [39]. More or less in contrast to our view on inclusion in NBS, patient organizations aim to include disorders that are “actionable” rather than “treatable” [36,50,51,52]. This broader view on the selection of disorders may result in including IMDs in which factors such as avoiding a long diagnostic odyssey or family planning might be important [53]. Although this discussion is ongoing and beyond the scope of the present study, the importance of clear definitions for those terms should not be underestimated, and defining the spectrum of “actionable”, “treatable”, and “curable” will remain challenging.

### 4.2. Consensus on the Selected Genes from the Literature Review

The genes related to IMDs in the current NBS elicited discussion. In retrospect, some genes, especially the incidental findings in the current NBS, should not have been automatically accepted in our final list, as their treatability is disputable. We corrected for this by carrying out a second review of all the genes in the current NBS [Table 1], as described in Methods Section 2.2.2 and Figure 1. This is a valuable lesson that illustrates the complexity of evaluating genes. In Appendix D, we elaborate on the genes involved.

The inclusion of some of the remaining 1397 genes was heavily disputed. Nineteen genes were excluded because the treatment options are still experimental, albeit promising. These genes could become interesting candidates for inclusion in the near future. Furthermore, the process of reviewing genes was not always straightforward. For some genes, there were both reasons for and against inclusion. This led to 43 genes [Appendix B] in which there was doubt or disagreement between the reviewers in the core team. We decided to not include these genes in the current list but to keep them in mind for future endeavors. Some convincing reports or pilot studies specifically advocated for the inclusion of an IMD in NBS, with CAD trifunctional protein deficiency (*CAD*, MIM *141010, #616457) [54] being a typical example of this.

### 4.3. Considerations for the Final List of Genes

The difficulty of reaching a consensus on which disorders should be included is exemplified by the fact that discussions were still ongoing even after the three rounds of meetings of the project group. This is partly explained by the challenge of defining treatability, but it can also reflect that some project group members have more (unpublished) knowledge about certain genes or disorders because of their role in a Center of Expertise in the Netherlands. Even after the list of 100 genes moved to the NGS4fNBS study [26], members of the project group argued against including the following IMDs: carnitine palmitoyltransferase 2 deficiency (CPT2) (*CPT2*, MIM *600650, #614212, #600649, #608836, #255110), carnitine-acylcarnitine translocase deficiency (CACT) (*SLC25A20*, MIM *613698, #212138), and mitochondrial acetoacetyl-CoA thiolase deficiency (BKT) (*ACAT1*, MIM *607809, #203750). CPT2 and CACT deficiency were questioned due to uncertainties in the natural course of these disorders. This opinion was underlined by negative advice for CPT2 deficiency from the Dutch Health Council, based on the large phenotypic variation [34], and this was seconded in very recent negative advice by the ANS-IMD (June 2024) based on the consideration that all (Dutch) patients found were adults. The ANS-IMD also advised negatively on BKT, based on a lack of clarity about the biomarker, and on CACT because it is often fatal. Therefore, in retrospect, CPT2 and CACT deficiency should not have been included in the list of 100 genes. In Appendix E, we discuss other debates.

This ongoing debate clearly shows that, for rare disorders, more data are needed to provide a more solid basis for the decision-making process about treatability, as well as for other criteria. We therefore encourage the publication of case studies and the creation of databases on treatability. It also shows the importance of keeping a record of the discussions and a clear formulation of the reasons for inclusion or exclusion.

### 4.4. Limitations

A limitation of this study is the risk of evidence selection bias. This bias could have occurred if our literature reviews did not identify all available evidence on treatability, and the fact is that the review process remains, to some degree, subjective. Our study also shows that it is hard to judge every disorder and the associated genes by the same standards, even when strict inclusion and exclusion criteria are formulated and used. By conducting this literature review with three researchers independently as a first step and then discussing the results with experts in the field, we aimed to minimize the risk of bias. However, as the last meeting illustrated, remarks can still be made even after an extensive literature review and a second review. For example, the treatment of TANGO2 deficiency, which presents with metabolic encephalopathy and arrhythmias, consists of avoiding the metabolic crisis by avoiding fasting or illness. This treatment strategy is considered sufficient to include MCAD (*ACADM*, MIM *607008 #201450) [55] in NBS, but not (yet) sufficient to include TANGO2 deficiency. Most of the difference here is a lack of understanding and evidence of the natural course of TANGO2 deficiency [56]. This illustrates how hard it is, even for extremely rare IMDs with comparable treatability, to obtain sufficient scientific evidence to fulfill all the W&J and Andermann criteria [31,32,33].

Another limitation was being consistent in the selection of specific groups of IMDs. This inconsistency arose because disorders not within the field of IMDs were included arbitrarily based on the experience of the project team. For example, Wilson’s disease (*ATP7B*, MIM *606882, #277900), cystinosis (*CTNS*, MIM *606272, #219800 #219900, #219750), and adenosine deaminase 1 deficiency (*ADA*, MIM *608958, #102700) were included since the project and core teams felt experienced enough to assess their treatability, while the combined project and core team felt less secure about chylomicron retention disease (*SAR1B*, MIM *607690, #246700) and mineralocorticoid receptor deficiency (*NR3C2*, MIM *600983, #605115, #177735). For that reason, we also excluded all disorders in steroid metabolism, e.g., congenital adrenal hyperplasia (CAH), steroid 21-hydroxylase deficiency (*CYP21A2*, MIM *613815, #201910), 11-beta-hydroxylase deficiency (*CYP11B1*, MIM *610613, #202010, #103900), 17-alpha-hydroxylase/17,20-lyase deficiency (*CYP17A1*, MIM *609300, #202110), cholesterol desmolase (*CYP11A1*, MIM *118485, #613743), and 3-beta-hydroxysteroid dehydrogenase (*HSD3B2*, MIM *613890, #201810). In the future, we would like to extend our list with monogenetic inherited disorders from various fields of medicine that are treatable and fulfill the other W&J [32,33] and Anderman criteria [31]. It can be expected that these criteria also need further development to become a fully transparent process.

Lastly, we did not address epigenetic and modifier genes. In some IMDs, epigenetic changes may contribute to the phenotypic variability of disorders. For example, a variant in *PRDX1* (MIM *176763, #277400) was found to cause an epimutation in the promotor of *MMACHC* (MIM *609831 #277400), leading to decreased expression of *MMACHC* thought to contribute to the phenotype [57,58]. Direct links between modifier genes and the clinical heterogenicity of IMDs are still under investigation [57], but these genes could be interesting to add in the future. For now, this further reinforces how difficult it is to assess every IMD by the same standards.

### 4.5. Applicability of Our Final List

We encourage others to use our list of 100 genes for further research and discussions into both the eligibility of IMDs and the applicability of NGS as a first- or second-tier strategy in NBS programs worldwide. We have included our list with each gene and its reason for exclusion in Appendix A and Appendix B to encourage others to constructively explore, investigate, and join us in the search for a universal—and transparent—list of disorders eligible for NBS and to learn from our challenges. For now, this 100-gene list includes genes for treatable IMDs, which may change with time due to new evidence. Options to expand this list with a separate list of genes for actionable disorders are also currently being investigated by our research group, taking into account recent suggestions for genes made by other groups [16,59,60,61] and consortia such as the International Consortium on Newborn Sequencing (ICoNS).

An important note on the applicability of our 100-gene list is that it is meant to be adaptable. In addition to the limited treatment options for some IMDs, one of the biggest challenges when including genes related to (ultra-)rare disorders is limited evidence for the pathogenicity of genetic variants. Using NGS as a first-tier screening will inevitably result in the detection of variants of unknown significance (VUSes). To investigate the pathogenicity of these variants, follow-up with biochemical tests is needed, and the availability of such a test should be taken into account when deciding if a gene should be included. When a test is available, other aspects like costs, test duration, and samples needed for testing should also be considered when deciding if the test would be feasible in an NBS setting. In addition, the 100 genes we identified were selected because the benefits of inclusion in NBS outweigh the disadvantages. However, other disorder-specific factors remained important because we screen the “genotype” to treat the phenotype, and the phenotype cannot always be predicted at the moment of screening. There is a consensus that IMDs with a severe and early onset should be included in NBS, but for mild phenotypes or disorders that may not present symptomatically during (young) infancy, or have both early and late-onset phenotypes, consensus on the appropriate strategy is still lacking [62,63,64]. An example of dealing with these uncertainties was Pompe’s disease (*GAA*, MIM *606800, #232300), for which treatment options are available for infantile Pompe patients. However, the majority of Pompe patients have a late-onset form, with first symptoms often in adulthood. As the two phenotypic groups cannot be accurately discriminated at the level of the individual patient, the disorder did not qualify for inclusion in our gene panel even when our focus was on treatability only. The uncertainty about the age of clinical presentation may result in a clear risk of unintended overtreatment, creating “patients in waiting” and causing unnecessary anxiety in parents, which might outweigh the advantages of screening for a specific IMD [63,64].

Furthermore, a major topic of discussion on the eligibility of IMDs for NBS remains the inclusion of (ultra-)rare disorders for which there is currently only very limited evidence of treatment. There is an intriguing interplay between introducing a disorder into NBS and the need to demonstrate the effectiveness of early treatment in pre-symptomatic diagnoses compared to treatment initiated after symptoms appear. Introducing a disorder into NBS can be a lengthy process for various reasons. However, piloting within NBS programs could be a valuable strategy for diseases with promising treatments still in trials, even before FDA or EMA approval, especially when the main barrier to inclusion is the lack of an adequate biomarker. This approach is exemplified by the introduction of NBS for Spinal Muscular Atrophy in the USA, where pilot programs were initiated before FDA approval of a key treatment [65]. An advantage of NGS as first-tier screening in NBS is that new genes can easily be added to the screening panel, without requiring entirely new methods for each new disorder, thereby accelerating the process. Including these IMDs in NBS thus remains controversial. However, without evidence from NBS, it could take decades of research before these disorders might be considered eligible. Including them could therefore help strengthen the evidence in favor (or against) their treatability. Therefore, once all W&J and Andermann criteria [31,32,33] have been met, and it is decided to screen for a specific disorder at the population level, we recommend that this selection be re-evaluated regularly. Computational tools for NGS can also aid in improving these gene panels. For now, we have decided not to include these ultra-rare disorders in the final list because the evidence on the benefit–harm ratio was unclear.

## 5. Conclusions

We used a systematic and transparent method to establish a list of 100 genes, associated with 95 IMDs, eligible for a genetic NBS. This method was successful for many genes, but we also faced challenges. In particular, the concept of “treatability” led to discussion. To define “treatability” in the light of NBS as clearly as possible, the development of standardized treatability scores is essential, preferably in an international setting. Such scores could prevent needless repetition of projects like ours and may help move the community towards a list of (internationally accepted) genes, with transparent reasons for inclusion and exclusion. At the same time, it is important to take into account not only the medical perspective, but also ethical, societal, governmental, and parental opinions.

Before the implementation of this list of 100 genes in NBS programs, the other W&J and Andermann criteria should be met and—as addressed above—some issues need to be discussed again. Our list is meant to be adaptable, and we invite the reader to join us in the search for a universal list of disorders eligible for an NGS-based NBS, paying attention to transparency.

## Figures and Tables

**Table 1:** Overview of Inherited Metabolic Disorders (IMD) and their associated genes in the Dutch Newborn Screening, as found by a biomarker-based first-tier screening.

IMD in the Dutch Newborn Screening	Associated Gene(s)	MIM	Secondary Findings IMD Due to Abnormal Biomarkers	Associated Gene(s)	MIM
1. Adenosine deaminase 1 deficiency (ADA SCID) as cause of severe combined immunodeficiency syndrome (SCID)	*ADA* *	*608958 #102700	1. Autosomal recessive GTP cyclohydrolase 1 deficiency	*GCH1*	*600225 #128230 #233910
2. Adrenoleukodystrophy	*ABCD1*	*300371 #300100	2. Dihydropteridine reductase deficiency	*QDPR*	*612676 #261630
3. Biotinidase deficiency	*BTD*	*609019 #253260	3. DNAJC12 deficiency	*DNAJC12*	*606060 #617384
4. Carnitine palmitoyl deficiency type 1	*CPT1A*	*600528 #255120	4. Flavin adenine dinucleotide synthetase deficiency	*FLAD1* ***	*610595 #255100
5. Galactokinase deficiency	*GALK1*	*604313 #230200	5. Maleylacetoacetate isomerase deficiency	*GSTZ1* ***	*603758 #617596
6. Galactosemia	*GALT*	*606999 #230400	6. Methylmalonacidemia **	*LMBRD1* *** SUCLA2 ***, *SUCLG1* ***, *MLYCD* ***, *ACSF3* ***	*612625 #277380, *603921 #612073, *611224 #245400, *606761 #248360 *614245 #614265
7. Glutaric aciduria type 1	*GCDH*	*608801 #231670	7. Methylmalonic acidemia with homocystinuria, combined **	*PRDX1* ***, *ABCD4* ***, *HCFC1* ***, *THAP11 (interacts with HCFC1)* ***, *TCN2*, *CD320* ***, *CBLIF* ***, *CUBN* ***, *AMN*, *ZNF143* ***	*176763 #277400 *603214 #614857, *300019 #309541, *609119, *613441 #275350, *606475 #613646, *609342 #261000, *602997 #261100 #618884, *605799 #618882, *603433
8. HMG-CoA lyase deficiency	*HMGCL*	*613898 #246450	8. Mitochondrial acetoacetyl-CoA thiolase deficiency	*ACAT1*	*607809 #203750
9. Isovaleric aciduria	*IVD*	*607036 #243500	9. Multiple acyl-CoA dehydrogenase deficiency	*ETFA*, *ETFB*, *ETFDH*	*608053 #231680, *130410 #231680, *231675 #231680
10. Maple syrup urine disease	*DBT*, *BCKDHA*, *BCKDHB*	*248610 #620699, *608348 #248600, *248611 #620698	10. Primary carnitine deficiency	*SLC22A5*	*603377 #212140
11. Medium-chain acyl-CoA dehydrogenase deficiency	*ACADM*	*607008 #201450	11. Pterin-4-a-Carbinolamine Dehydratase deficiency	*PCBD1*	*126090 #264070
12. Methylmalonacidemia **	*MMUT*, *MMAA*, *MMAB* ***, *MMACHC*, *MMADHC*, *MCEE*	*609058 #251000, *607481 #251100, *607568 #251110, *609831 #277400, *611935 #277410, *608419 #251120	12. Riboflavin transporter deficiencies (synonym: Brown–Vialetto–van Laere syndrome type 1 and 2)	*SLC52A1* ***, *SLC52A2*, *SLC52A3*	*607883 #615026, *607882 #614707, *613350 #211500 #211530
13. Multiple CoA carboxylase deficiency	*HLCS*	*609018 #253270	13. 2-Methyl-3-hydroxybutyryl-CoA Dehydrogenase deficiency	*HSD17B10* ***	*300256 #300438
14. Phenylketonuria	*PAH*	*612349 #261600	14. 3-Methylglutaconyl-CoA hydratase deficiency	*AUH* ***	*600529 #250950
15. Propionic acidemia	*PCCA*, *PCCB*	*232000 #606054, *232050 #606054	15. 6-Pyruvoyl-tetrahydropterin synthase deficiency	*PTS*	*612719 #261640
16. Trifunctional protein deficiency/ long-chain hydroxyacyl-CoA dehydrogenase deficiency	*HADHA*, *HADHB* ***	*600890 #609016 #609015, *143450 #620300			
17. Tyrosinemia type 1	*FAH*	*613871 #276700			
18. Very-long-chain acyl-CoA dehydrogenase deficiency	*ACADVL*	*609575 #201475			
19. Mucopolysaccharidosis type 1	*IDUA*	*252800 #607014 #607015 #607016			
20. 3-Methylcrotonyl-CoA carboxylase deficiency	*MCCC1*, *MCCC2*	*609010 #210200, *609014 #210210			

* The Netherlands currently screens for Omenn’s syndrome (MIM #603554) and atypical/“leaky” SCID. Fifteen percent of SCID patients have ADA deficiency (MIM *608958 #102700), also an IMD, as the underlying genetic cause. Other non-IMDs in the Dutch NBS (per 1 January 2024) are congenital adrenal hyperplasia, congenital hypothyroidism, cystic fibrosis, Hemoglobin H disease (alpha thalassemia), sickle cell disease, spinal muscular atrophy, and bèta-thalassemia, major [4,12,13]. ** The governmental guidance in the Netherlands is rather unclear on the precise definition of methylmalonic acidemia to be included in NBS [13]. The presumed secondary findings of methylmalonic acidemia are depicted in the right column, numbers 6 and 7. *** Genes in blue are not included in our final list of treatable IMDs (see Discussion Section 4.2).

**Table 2:** Overview of the 100 genes (corresponding to 95 phenotypic IMDs in OMIM) eligible for newborn screening based only on treatability.

*ABCD1* *	*BAAT*	*FBP1*	*IDUA* *	*PTS* **
*ACADM* *	*BCKDHA* *	*FOLR1*	*IVD* *	*QDPR* **
*ACADVL* *	*BCKDHB* *	*G6PC*	*LPL*	*SI*
*ACAT1* **	*BCKDK*	*GALK1* *	*MCCC1* *	*SLC19A3*
*ADA* *	*BTD* *	*GALT* *	*MCCC2* *	*SLC22A5* **
*AGL*	*CA5A*	*GAMT*	*MCEE* **	*SLC25A15*
*AGXT*	*CAD*	*GATM*	*MMAA* *	*SLC25A20*
*AHCY*	*CBS*	*GBA*	*MMACHC* *	*SLC2A1*
*AKR1D1*	*CPS1*	*GCDH* *	*MMADHC* *	*SLC2A2*
*AKT2*	*CPT1A* *	*GCH1* **	*MMUT* *	*SLC37A4*
*ALDH7A1*	*CPT2*	*GCK*	*NAGS*	*SLC40A1*
*ALDOB*	*CTNS*	*GLUD1*	*OAT*	*SLC46A1*
*AMN* **	*CTPS1*	*GPIHBP1*	*OTC*	*SLC52A2* **
*APOC2*	*CYP27A1*	*GYS2*	*OXCT1*	*SLC52A3* **
*APOE*	*DBT* *	*HADHA* *	*PAH* *	*SLC5A1*
*ARG1*	*DNAJC12* **	*HJV*	*PCBD1* **	*TAT*
*ARSA*	*ETFA* **	*HLCS* *	*PCCA* *	*TCN2* **
*ASL*	*ETFB* **	*HMGCL* *	*PCCB* *	*TH*
*ASS1*	*ETFDH* **	*HMGCS2*	*PGM1*	*TPK1*
*ATP7B*	*FAH* *	*HSD3B7*	*PNP*	*TTPA*

* Included in the current NBS as a primary target, also depicted in the darkest shade of blue. ** Included in the current NBS or secondary finding, also depicted in a medium shade of blue. IMDs that are not in the current NBS are depicted in the lightest shade of blue.

## Data Availability

Data are contained within the article.

## Appendix A

**Table A1:** Overview of 100 genes associated with 95 Inherited Metabolic Disorders eligible for a genetic Newborn Screening based on treatability.

Inherited Metabolic Disorders	Associated Gene	MIM Phenotype	MIM Gene/ Locus
1. 3-hydroxy-3-methylglutaryl-CoA lyase deficiency	*HMGCL*	246450	613898
2. 3-Methylcrotonyl-CoA carboxylase 1 deficiency (synonym: 3-methylcrotonylglycinuria type 1)	*MCCC1*	210200	609010
3. 3-Methylcrotonyl-CoA carboxylase 2 deficiency (synonym: 3-methylcrotonylglycinuria type 2)	*MCCC2*	210210	609014
4. 3β-Hydroxy-Δ5-C27-steroid oxidoreductase deficiency	*HSD3B7*	607765	607764
5. 6-Pyruvoyl-tetrahydropterin synthase deficiency	*PTS*	261640	612719
6. Adenosine deaminase 1 deficiency	*ADA*	102700	608958
7. AKT2 superactivity (synonym: hypoinsulinemic hypoglycemia with hemihypertrophy)	*AKT2*	240900	164731
8. Alanine-glyoxylate aminotransferase deficiency (synonym: Primary hyperoxaluria type 1)	*AGXT*	259900	604285
9. Aldolase B deficiency (synonym: hereditary fructose intolerance)	*ALDOB*	229600	612724
10. Amnionless deficiency (synonym: Imerslund-Gräsbeck disease, Norwegian type)	*AMN*	618882	605799
11. Apolipoprotein C2 deficiency	*APOC2*	207750	608083
12. Apolipoprotein E deficiency (synonym: dysbetalipoproteinemia)	*APOE*	617347	107741
13. Arginase deficiency (synonym: argininemia)	*ARG1*	207800	608313
14. Arginineglycine amidinotransferase (AGAT) deficiency	*GATM*	612718, 134600	602360
15. Argininosuccinate lyase deficiency	*ASL*	207900	608310
16. Argininosuccinate synthetase deficiency (synonym: citrullinemia type 1)	*ASS1*	215700	603470
17. Arylsulfatase A deficiency (synonym: metachromatic leukodystrophy)	*ARSA*	250100	607574
18. Autosomal recessive GTP cyclohydrolase 1 deficiency	*GCH1*	233910, 128230	600225
19. Bile acid-CoA:amino acid N-acyltransferase deficiency	*BAAT*	619232	602938
20. Biotinidase deficiency	*BTD*	253260	609019
21. Branched-chain ketoacid dehydrogenase E1α deficiency (synonym: maple syrup urine disease type 1a, MSUD1a)	*BCKDHA*	248600	608348
22. Branched-chain ketoacid dehydrogenase E1β deficiency (synonym: maple syrup urine disease type 1b, MSUD1b)	*BCKDHB*	248600	248611
23. Branched-chain ketoacid dehydrogenase kinase deficiency	*BCKDK*	614923	614901
24. CAD trifunctional protein deficiency	*CAD*	616457	114010
25. Carbamoyl phosphate synthetase 1 deficiency	*CPS1*	237300	608307
26. Carbonic anhydrase VA deficiency	*CA5A*	615751	114761
27. Carnitine palmitoyltransferase 1A deficiency	*CPT1A*	255120	600528
28. Carnitine palmitoyltransferase 2 deficiency	*CPT2*	600649, 608836, 255110	600650
29. Carnitine-acylcarnitine translocase deficiency	*SLC25A20*	212138	613698
30. Congenital sucrase-isomaltase deficiency	*SI*	222900	609845
31. Copper-transporting ATPase β subunit deficiency (synonym: Wilson disease (WD))	*ATP7B*	277900	606882
32. CTP synthase 1 deficiency	*CTPS1*	615897	123860
33. Cystathionine β-synthase deficiency (synonym: classic homocystinuria)	*CBS*	236200	613381
34. Cystinosis	*CTNS*	219800, 219900, 219750	606272
35. Dihydrolipoyl transacylase deficiency (synonyms: maple syrup urine disease type 2 (MSUD 2), branched-chain ketoacid dehydrogenase E2 deficiency)	*DBT*	620699	248610
36. Dihydropteridine reductase deficiency	*QDPR*	248600	612676
37. DNAJC12 deficiency	*DNAJC12*	617384	606060
38. Electron transfer flavoprotein dehydrogenase deficiency (synonym: glutaric acidemia type 2C, multiple acyl-CoA dehydrogenase deficiency type 2C (MADD type 2C))	*ETFDH*	231680	231675
39. Electron transfer flavoprotein α subunit deficiency (synonym: glutaric acidemia type 2A, multiple acyl-CoA dehydrogenase deficiency type 2A (MADD type 2A))	*ETFA*	231680	608053
40. Electron transfer flavoprotein β subunit deficiency (synonym: glutaric acidemia type 2B, multiple acyl-CoA dehydrogenase deficiency type 2B (MADD type 2B))	*ETFB*	231680	130410
41. Ferroportin deficiency (synonym: hereditary hemochromatosis type 4)	*SLC40A1*	606069	604653
42. Folate receptor α deficiency (synonym: neurodegeneration due to cerebral folate transport deficiency)	*FOLR1*	613068	136430
43. Fructose-1,6-bisphosphatase deficiency	*FBP1*	229700	611570
44. Fumarylacetoacetase deficiency (synonym: Tyrosinemia type 1)	*FAH*	276700	613871
45. Galactokinase deficiency	*GALK1*	230200	604313
46. Galactose-1-phosphate uridylyltransferase deficiency (synonym: classic galactosemia)	*GALT*	230400	606999
47. Glucocerebrosidase deficiency (synonym: Gaucher disease type I, II, III, IIIC)	*GBA*	230800, 230900, 231000, 230105	606463
48. Glucokinase deficiency	*GCK*	606176, 602485, 125853, 125851	138079
49. Glucose transporter 2 deficiency (synonym: Fanconi-Bickel syndrome)	*SLC2A2*	227810	138160
50. Glucose-6-phosphatase deficiency (synonym: glycogen storage disease type 1a)	*G6PC*	232200	613742
51. Glucose-6-phosphate transporter deficiency (synonym: glycogen storage disease type 1b)	*SLC37A4*	232220, 232240, 619525	602671
52. GLUT1 deficiency	*SLC2A1*	606777, 612126, 608885, 601042	138140
53. Glutamate dehydrogenase superactivity (synonym: hyperinsulinism-hyperammonemia syndrome)	*GLUD1*	606762	138130
54. Glutaryl-CoA dehydrogenase deficiency (synonym: glutaric acidemia type 1)	*GCDH*	231670	608801
55. Glycogen debranching enzyme deficiency (synonyms: glycogen storage disease type 3, Cori-Forbes disease, limit dextrinosis)	*AGL*	232400	610860
56. GPIHBP1 deficiency	*GPIHBP1*	615947	612757
57. Guanidinoacetate methyltransferase deficiency (GAMT)	*GAMT*	612736	601240
58. Hemojuvelin deficiency	*HJV*	602390	608374
59. Hepatic glycogen synthase deficiency (synonym: glycogen storage disease type 0a)	*GYS2*	240600	138571
60. Holocarboxylase synthetase deficiency	*HLCS*	253270	609018
61. Homocystinuria, cblDv1 type	*MMADHC*	277410, 620953, 620952	611935
62. Intestinal sodium-glucose cotransporter 1 deficiency (synonym: glucose-galactose malabsorption)	*SLC5A1*	606824	182380
63. Isovaleryl-CoA dehydrogenase deficiency (synonym: isovaleric academia)	*IVD*	243500	607036
64. Lipoprotein lipase deficiency	*LPL*	238600, 144250	609708
65. Medium-chain acyl-CoA dehydrogenase deficiency (MCAD)	*ACADM*	201450	607008
66. Methylmalonic acidemia and homocystinuria, cblC type	*MMACHC*	277400	609831
67. Methylmalonic acidemia due to methylmalonyl-CoA epimerase deficiency	*MCEE*	251120	608419
68. Methylmalonic acidemia due to methylmalonyl-CoA mutase deficiency	*MMUT*	251000	609058
69. Methylmalonic acidemia, cblA type	*MMAA*	251100	607481
70. Mitochondrial 3-hydroxy-3-methylglutaryl-CoA synthase deficiency	*HMGCS2*	605911	600234
71. Mitochondrial acetoacetyl-CoA thiolase deficiency	*ACAT1*	203750	607809
72. Mitochondrial ornithine transporter deficiency (synonym: hyperornithinemia-hyperammonemia-homocitrullinuria syndrome)	*SLC25A15*	238970	603861
73. N-acetylglutamate synthase deficiency	*NAGS*	237310	608300
74. Ornithine aminotransferase deficiency (synonym: gyrate atrophy of choroid and retina)	*OAT*	258870	613349
75. Ornithine transcarbamylase deficiency	*OTC*	311250	300461
76. Phenylalanine hydroxylase deficiency (synonym: phenylketonuria)	*PAH*	261600	612349
77. Phosphoglucomutase 1 deficiency (PGM1-CDG)	*PGM1*	614921	171900
78. Primary carnitine deficiency	*SLC22A5*	212140	603377
79. Propionic acidemia due to propionyl-CoA carboxylase α subunit deficiency	*PCCA*	606054	232000
80. Propionic acidemia due to propionyl-CoA carboxylase β subunit deficiency	*PCCB*	606054	232050
81. Proton-coupled folate transporter deficiency (synonym: hereditary folate malabsorption)	*SLC46A1*	229050	611672
82. Pterin-4-α-carbinolamine dehydratase deficiency	*PCBD1*	264070	126090
83. Purine nucleoside phosphorylase deficiency	*PNP*	613179	164050
84. Riboflavin transporter 2 deficiency (synonym: Brown–Vialetto–van Laere syndrome type 1)	*SLC52A3*	211500, 211530	613350
85. Riboflavin transporter 3 deficiency (synonym: Brown–Vialetto–van Laere syndrome type 2)	*SLC52A2*	614707	607882
86. S-adenosylhomocysteine hydrolase deficiency	*AHCY*	613752	180960
87. Sterol 27-hydroxylase deficiency	*CYP27A1*	213700	606530
88. Succinyl-CoA:3-oxoacid-CoA transferase deficiency (SCOT deficiency)	*OXCT1*	245050	601424
89. Thiamine pyrophosphokinase deficiency	*TPK1*	614458	606370
90. Thiamine transporter 2 deficiency (synonym: biotin-thiamine-responsive basal ganglia disease)	*SLC19A3*	607483	606152
91. Transcobalamin II deficiency	*TCN2*	275350	613441
92. Trifunctional protein α subunit deficiency (LCHAD)	*HADHA*	609015, 609016	600890
93. Tyrosine aminotransferase deficiency (synonyms: tyrosinemia type 2; Richner–Hanhart syndrome)	*TAT*	276600	613018
94. Tyrosine hydroxylase deficiency	*TH*	605407	191290
95. Very long-chain acyl-CoA dehydrogenase deficiency (VLCAD)	*ACADVL*	201475	609575
96. X-linked adrenoleukodystrophy	*ABCD1*	300100	300371
97. α-aminoadipic semialdehyde dehydrogenase deficiency (synonym: pyridoxine-dependent epilepsy)	*ALDH7A1*	266100	107323
98. α-iduronidase deficiency	*IDUA*	607014, 607015, 607016	252800
99. α-tocopherol transfer protein deficiency (synonym: ataxia with isolated vitamin E deficiency)	*TTPA*	277460	600415
100. Δ4-3-oxosteroid 5β-reductase deficiency	*AKR1D1*	235555	604741

## Appendix B

**Table A2:** Overview of 1359 genes/genetic IMDs and their reason for exclusion.

**Genes excluded because of a lack of literature (*N* = 1003)**
1. No genotype–phenotype relation is known for the disorder (Total *N* = 71 and *N* = 15 disorders for which there was no phenotype/genotype relation)
*ACACB*, *ACMSD*, *AGMO*, *ALDH1L2*, *ALPI*, *ANPEP*, *APOO*, *ATP5PO*, *BBOX1*, *BLOC1S3*, *BLOC1S6*, *CCS*, *COX16*, *DTNBP1*, *DTYMK*, *DUT*, *EIF6*, *FAAH2*, *FA2H*, *FAM20B*, *GALNT14*, *GABRA6*, *GFUS*, *GNPNAT1*, *GON7*, *GYG2*, *HACD1*, *KMO*, *LAP3*, *LIPN*, *MAT2A*, *MCAT*, *MOCS3*, *MPST*, *NAPB*, *NDUFA8*, *NDUFAF7*, *NDUFB7*, *NME3*, *NPL*, *NT5E*, *OXA1L*, *PDPR*, *PDZK1IP1*, *PDE12*, *PHYKPL*, *PLIN5*, *PNPLA4*, *POLRMT*, *PRORP*, *RIC3*, *RNF31*, *RPS20*, *SAT1*, *SHPK*, *SLC10A1*, *SLC19A1*, *SLC27A5*, *SLC29A1*, *SORD*, *SORCS3*, *SQOR*, *STAP1*, *SUGCT*, *SV2A*, *TAF1A*, *TCN1*, *TLCD3B*, *TOMM70*, *UGCG*, *UQCRFS1*, *VPS4A*, *YRDC*.
2. Insufficient evidence for an (effective) treatment (*N* = 897, one IMD has multiple gene entries (not included))
*A4GALT*, *AARS1*, *AARS2*, *ABAT*, *ABCD3*, *ABHD12*, *ABHD5*, *ACACA*, *ACAD8*, *ACAD9*, *ACADSB*, *ACBD5*, *ACER3*, *ACO2*, *ACOX1*, *ACOX2*, *ACSL4*, *ACY1*, *ADA*, *ADA2*, *ADAR*, *ADAT3*, *ADSL*, *AFG3L2*, *AGA*, *AGK*, *AGPAT2*, *AGPS*, *AICDA*, *AIFM1*, *AIMP1*, *AIMP2*, *AK1*, *ALDH18A1*, *ALDH18A1*, *ALDH3A2*, *ALDH6A1*, *ALDOA*, *ALG1*, *ALG11*, *ALG12*, *ALG13*, *ALG2*, *ALG3*, *ALG6*, *ALG8*, *ALG9*, *AMACR*, *AMPD2*, *AMPD3*, *ANGPTL3*, *AP1B1*, *AP1S1*, *AP1S2*, *AP3B1*, *AP3B2*, *AP3D1*, *AP4B1*, *AP4E1*, *AP4M1*, *AP4S1*, *AP5Z1*, *APOC3*, *APOE*, *APOE*, *APOPT1*, *ARCN1*, *ARFGEF2*, *ARSG*, *ASAH1*, *ASAH1*, *ASNS*, *ASPA*, *ATAD3A*, *ATG5*, *ATIC*, *ATP5F1A*, *ATP5F1D*, *ATP5F1E*, *ATP5MD*, *ATP6AP1*, *ATP6AP2*, *ATP6V0A2*, *ATP6V1A*, *ATP6V1E1*, *ATP7A*, *ATP7A*, *ATP8A2*, *ATPAF2*, *AUH*, *B3GALNT2*, *B3GALT6*, *B3GAT3*, *B3GLCT*, *B4GALNT1*, *B4GALT1*, *B4GALT7*, *B4GAT1*, *BCAP31*, *BCAT2*, *BCS1L*, *BMS1*, *BOLA3*, *BPNT2*, *C12orf65*, *C1GALT1C1*, *C1QBP*, *CANT1*, *CARS1*, *CARS2*, *CAT*, *CCDC115*, *CEP89*, *CERS1*, *CERS2*, *CETP*, *CHAT*, *CHCHD10*, *CHKB*, *CHRNE*, *CHST14*, *CHST6*, *CHSY1*, *CLCN2*, *CLN3*, *CLN5*, *CLN6*, *CLN8*, *CLP1*, *CLPB*, *CLPP*, *CLPX*, *CLTC*, *CMPK2*, *CNDP1*, *COA3*, *COA5*, *COA6*, *COA7*, *COASY*, *COG1*, *COG2*, *COG4*, *COG4*, *COG5*, *COG6*, *COG7*, *COG8*, *COL4A3BP*, *COPA*, *COPB2*, *COQ4*, *COQ8B*, *COX10*, *COX14*, *COX15*, *COX20*, *COX4I1*, *COX4I2*, *COX5A*, *COX6A1*, *COX6A2*, *COX6B1*, *COX7B*, *COX8A*, *CPOX*, *CRAT*, *CRPPA*, *CSGALNACT1*, *CTSA*, *CTSC*, *CTSD*, *CTSF*, *CTSK*, *CYB5R3*, *CYP11B2*, *CYP11B2*, *CYP2U1*, *CYP4F22*, *CYP51A1*, *CYP7A1*, *CYP7B1*, *D2HGDH*, *DALRD3*, *DARS1*, *DARS2*, *DDC*, *DDHD1*, *DDHD2*, *DDOST*, *DEGS1*, *DGAT1*, *DGUOK*, *DHCR24*, *DHDDS*, *DHTKD1*, *DIABLO*, *DKC1*, *DMGDH*, *DNA2*, *DNAJC19*, *DNAJC21*, *DNM1*, *DNM2*, *DOLK*, *DPAGT1*, *DPM1*, *DPM2*, *DPM3*, *DPYD*, *DPYS*, *DSE*, *DYM*, *DYNC1H1*, *EARS2*, *EBP*, *EBP*, *ECHS1*, *EHHADH*, *ELAC2*, *ELOVL1*, *ELOVL4*, *ELOVL4*, *ELOVL5*, *ELP1*, *ELP2*, *EMC1*, *EMG1*, *EPG5*, *EPM2A*, *EPRS1*, *ERAL1*, *EXT2*, *EXTL3*, *FAR1*, *FARS2*, *FARSA*, *FARSB*, *FASTKD2*, *FBXL4*, *FDFT1*, *FDX2*, *FDXR*, *FECH*, *FH*, *FH*, *FIG4*, *FIG4*, *FKRP*, *FKTN*, *FOXRED1*, *FTL*, *FTL*, *FTSJ1*, *FUCA1*, *FUK*, *FUT8*, *G6PC3*, *GAA*, *GABBR2*, *GABRA1*, *GABRB1*, *GABRB2*, *GABRB3*, *GABRD*, *GABRG2*, *GAD1*, *GALNS*, *GALNT3*, *GANAB*, *GARS1*, *GATC*, *GBA2*, *GCK*, *GCLC*, *GDAP1*, *GFER*, *GFM1*, *GFM2*, *GGPS1*, *GGT1*, *GK*, *GLA*, *GLB1*, *GLB1*, *GLRA1*, *GLRB*, *GLYCTK*, *GM2A*, *GMPPA*, *GMPPB*, *GNE*, *GNE*, *GNMT*, *GNPAT*, *GNPTAB*, *GNPTG*, *GORAB*, *GOSR2*, *GPAA1*, *GPD1*, *GPHN*, *GPI*, *GPT2*, *GPX4*, *GRIA2*, *GRIA3*, *GRIA4*, *GRID2*, *GRM1*, *GRM1*, *GSR*, *GSS*, *GTPBP3*, *GUF1*, *GYS1*, *HAAO*, *HADH*, *HAO1*, *HARS1*, *HARS2*, *HCCS*, *HEPHL1*, *HEXA*, *HEXB*, *HFE*, *HIBADH*, *HIBCH*, *HK1*, *HK1*, *HMOX1*, *HPD*, *HPGD*, *HPRT1*, *HPS3*, *HPS5*, *HPS6*, *HS6ST1*, *HS6ST2*, *HSD17B4*, *HSPA9*, *HSPD1*, *HSPE1*, *HTRA2*, *HYAL1*, *IARS1*, *IARS2*, *IBA57*, *IDH1*, *IDH2*, *IDH3A*, *IDH3B*, *IFIH1*, *IL1RAPL1*, *IMPDH1*, *INPP5E*, *INPP5K*, *INPPL1*, *INS*, *INS*, *INSR*, *ISCA1*, *ISCA2*, *ISCU*, *ITPA*, *ITPR1*, *KARS1*, *KARS1*, *KCTD7*, *KDSR*, *KIF1A*, *KIF5C*, *KYNU*, *L2HGDH*, *LAGE3*, *LAMP2*, *LARGE1*, *LARS1*, *LARS2*, *LCAT*, *LDHD*, *LFNG*, *LIAS*, *LIPA*, *LIPC*, *LIPE*, *LIPT1*, *LIPT2*, *LONP1*, *LPA*, *LPIN1*, *LPIN2*, *LRPPRC*, *LSS*, *LTC4S*, *LYRM4*, *LYRM7*, *LYST*, *MAGT1*, *MAN1B1*, *MAN2B2*, *MANBA*, *MAOA*, *MARS1*, *MARS2*, *MAT1A*, *MBOAT7*, *MBTPS1*, *MCOLN1*, *MDH1*, *MDH2*, *MDH2*, *MECR*, *MFF*, *MFN2*, *MFSD2A*, *MFSD8*, *MGAT2*, *MGME1*, *MICOS13*, *MICU1*, *MICU2*, *MIEF2*, *MIPEP*, *MMAB*, *MOCOS*, *MOCS2*, *MOGS*, *MPC1*, *MPDU1*, *MPI*, *MPV17*, *MRM2*, *MRPL12*, *MRPL24*, *MRPL3*, *MRPL44*, *MRPS14*, *MRPS16*, *MRPS2*, *MRPS22*, *MRPS23*, *MRPS25*, *MRPS28*, *MRPS34*, *MRPS7*, *MSMO1*, *MSTO1*, *MT-ATP6*, *MT-ATP8*, *MT-CO1*, *MT-CO2*, *MT-CO3*, *MT-CYB*, *MT-ND1*, *MT-ND2*, *MT-ND3*, *MT-ND4*, *MT-ND4L*, *MT-ND5*, *MT-ND6*, *MT-RNR1*, *MT-RNR2*, *MT-TA*, *MT-TC*, *MT-TD*, *MT-TE*, *MT-TF*, *MT-TG*, *MT-TH*, *MT-TI*, *MT-TK*, *MT-TL1*, *MT-TL2*, *MT-TM*, *MT-TN*, *MT-TP*, *MT-TQ*, *MT-TR*, *MT-TS1*, *MT-TS2*, *MT-TT*, *MT-TV*, *MTTW*, *MTTY*, *MTFMT*, *MTHFD1*, *MTM1*, *MTMR2*, *MTO1*, *MTPAP*, *MTTP*, *MVK*, *MVK*, *NADSYN1*, *NAGA*, *NANS*, *NARS2*, *NAT8L*, *NAXD*, *NAXE*, *NBAS*, *NDST1*, *NDUFA1*, *NDUFA10*, *NDUFA11*, *NDUFA12*, *NDUFA13*, *NDUFA2*, *NDUFA4*, *NDUFA6*, *NDUFA9*, *NDUFAF1*, *NDUFAF2*, *NDUFAF3*, *NDUFAF4*, *NDUFAF5*, *NDUFAF6*, *NDUFAF8*, *NDUFB10*, *NDUFB11*, *NDUFB3*, *NDUFB8*, *NDUFB9*, *NDUFC2*, *NDUFS1*, *NDUFS2*, *NDUFS3*, *NDUFS4*, *NDUFS6*, *NDUFS7*, *NDUFS8*, *NDUFV1*, *NDUFV2*, *NEPRO*, *NEU1*, *NFS1*, *NFU1*, *NGLY1*, *NHLRC1*, *NMNAT1*, *NNT*, *NPC1*, *NPC2*, *NSDHL*, *NSUN2*, *NSUN3*, *NT5C3A*, *NUBPL*, *NUDT15*, *NUS1*, *OCRL*, *OGDH*, *OGT*, *OPA1*, *OPA3*, *OPLAH*, *OSGEP*, *PAICS*, *PAM16*, *PANK2*, *PARS2*, *PC*, *PCK1*, *PCK2*, *PCSK9*, *PCYT1A*, *PCYT1A*, *PCYT2*, *PDK3*, *PEPD*, *PET100*, *PET117*, *PEX1*, *PEX10*, *PEX11B*, *PEX12*, *PEX13*, *PEX14*, *PEX16*, *PEX19*, *PEX2*, *PEX26*, *PEX3*, *PEX5*, *PEX5*, *PEX6*, *PEX7*, *PGAM2*, *PGAP1*, *PGAP2*, *PGAP3*, *PGM3*, *PHYH*, *PI4K2A*, *PI4KA*, *PIGB*, *PIGC*, *PIGG*, *PIGH*, *PIGK*, *PIGL*, *PIGN*, *PIGP*, *PIGQ*, *PIGS*, *PIGT*, *PIGU*, *PIGV*, *PIGW*, *PIGY*, *PIK3C2A*, *PIK3CA*, *PIK3CD*, *PIK3R1*, *PIK3R2*, *PIK3R5*, *PIKFYVE*, *PIP5K1C*, *PISD*, *PITRM1*, *PLA2G4A*, *PLA2G6*, *PLCB1*, *PLCB3*, *PLCB4*, *PLCD1*, *PLCE1*, *PLCG2*, *PLIN1*, *PLPBP*, *PMPCA*, *PMPCB*, *PNKD*, *PNPLA2*, *PNPLA6*, *PNPLA8*, *PNPO*, *PNPT1*, *POGLUT1*, *POLG*, *POLG2*, *POLR1A*, *POLR1B*, *POLR1C*, *POLR1C*, *POLR1D*, *POLR3A*, *POLR3A*, *POLR3B*, *POMGNT1*, *POMGNT2*, *POMK*, *POMT1*, *POMT2*, *POP1*, *PPCS*, *PPT1*, *PRKAG2*, *PRODH*, *PSAP*, *PSAP*, *PSAP*, *PSAP*, *PTCD3*, *PTDSS1*, *PTEN*, *PTRH2*, *PUS1*, *PUS3*, *PYCR1*, *PYCR2*, *QARS1*, *QRSL1*, *RAB18*, *RAB23*, *RAB3GAP1*, *RAB3GAP2*, *RAB7A*, *RARS1*, *RARS2*, *RBCK1*, *RBSN*, *RFT1*, *RFX6*, *RMND1*, *RMRP*, *RNASEH1*, *RNASEH2A*, *RNASEH2B*, *RNASEH2C*, *RNASET2*, *RPIA*, *RPL10*, *RPS23*, *RRM2B*, *RTN4IP1*, *RUBCN*, *RXYLT1*, *SACS*, *SAMHD1*, *SARS1*, *SARS2*, *SBF1*, *SBF2*, *SC5D*, *SCARB1*, *SCARB2*, *SCO1*, *SCO2*, *SCP2*, *SCYL1*, *SCYL2*, *SDHAF1*, *SDHB*, *SDHD*, *SECISBP2*, *SELENOI*, *SEPSECS*, *SERAC1*, *SFXN4*, *SGMS2*, *SGPL1*, *SLC10A2*, *SLC11A2*, *SLC13A3*, *SLC13A5*, *SLC17A5*, *SLC1A1*, *SLC1A2*, *SLC1A3*, *SLC1A4*, *SLC22A12*, *SLC25A1*, *SLC25A10*, *SLC25A11*, *SLC25A12*, *SLC25A21*, *SLC25A22*, *SLC25A24*, *SLC25A26*, *SLC25A3*, *SLC25A38*, *SLC25A4*, *SLC25A42*, *SLC25A46*, *SLC26A1*, *SLC28A1*, *SLC29A3*, *SLC2A10*, *SLC2A9*, *SLC30A9*, *SLC33A1*, *SLC35A1*, *SLC35A1*, *SLC35A3*, *SLC35C1*, *SLC38A8*, *SLC39A13*, *SLC45A1*, *SLC5A2*, *SLC5A7*, *SLC6A17*, *SLC6A19*, *SLC6A2*, *SLC6A3*, *SLC6A5*, *SLC6A9*, *SLC7A14*, *SLC7A3*, *SLC7A5*, *SLC9A7*, *SLCO2A1*, *SMPD1*, *SMPD4*, *SMS*, *SNAP25*, *SNORD118*, *SNX14*, *SPATA5*, *SPG11*, *SPG20*, *SPG7*, *SPNS2*, *SPTLC1*, *SPTLC2*, *SRD5A3*, *SSBP1*, *SSR3*, *SSR4*, *ST3GAL3*, *ST3GAL5*, *STAT2*, *STT3A*, *STT3B*, *STX11*, *STX1B*, *STXBP1*, *STXBP2*, *SUMF1*, *SURF1*, *SYN1*, *SYNJ1*, *SYT1*, *SYT14*, *SYT2*, *TACO1*, *TALDO1*, *TANGO2*, *TARS1*, *TARS2*, *TAZ*, *TBXAS1*, *TCOF1*, *TECPR2*, *TECR*, *TFAM*, *TFRC*, *THG1L*, *TIMM22*, *TIMM50*, *TIMM8A*, *TIMMDC1*, *TIMMDC1*, *TK2*, *TKFC*, *TKT*, *TMEM126A*, *TMEM126B*, *TMEM165*, *TMEM173*, *TMEM70*, *TMPRSS6*, *TOP3A*, *TOR1A*, *TP53RK*, *TPI1*, *TPMT*, *TPP1*, *TPRKB*, *TRAK1*, *TRAPPC11*, *TRAPPC12*, *TRAPPC4*, *TRAPPC6B*, *TRAPPC9*, *TREX1*, *TRIT1*, *TRMT1*, *TRMT10C*, *TRMT5*, *TSEN15*, *TSEN2*, *TSEN34*, *TSFM*, *TTC19*, *TUFM*, *TUSC3*, *TWNK*, *TXN2*, *TXNRD2*, *TYR*, *UBTF*, *UGDH*, *UGP2*, *UGT1A1*, *UNC13D*, *UPB1*, *UQCC2*, *UQCC3*, *UQCRB*, *UQCRC2*, *UQCRQ*, *UROC1*, *VAPB*, *VARS1*, *VARS2*, *VIPAS39*, *VLDLR*, *VPS11*, *VPS13B*, *VPS33A*, *VPS33B*, *VPS45*, *WARS1*, *WARS2*, *WDR4*, *WDR45*, *XDH*, *XPNPEP3*, *XYLT1*, *XYLT2*, *YARS1*, *YARS2*, *YIF1B*, *YME1L1*, *ZFYVE26*
3. Extremely rare disorders (or frequency yet unknown), only described in 5 or fewer patients (*N* = 18)
*ACAT2*, *CYB5A*, *DBH*, *ENO3*, *FCSK*, *FTH1*, *GATB*, *GLS*, *GLS*, *GYG1*, *HYKK*, *NADK2*, *NFE2L2*, *ODC1*, *PSAT1*, *PSPH*, *SLC7A2*, *TRAPPC2L*
**Genes excluded on the basis of treatability (*N* = 177)**
4. Contradicting literature on the treatment outcome (*N* = 6)
*GRIN1*, *GRIN2A*, *GRIN2B*, *GRIN2D*, *KCNJ11*, *SLC18A2*
5. Poor prognosis (despite treatment) (*N* = 15)
*ABCC6*, *DNM1L*, *ENPP1*, *ETHE1*, *GALC*, *GLUL*, *HSD17B10*, *KCNJ11*, *NSDHL*, *RAB27A*, *SLC25A20*, *SLC35D1*, *SNAP29*, *SUOX*, *VAMP1*
6. Variable phenotype, with one phenotype’s treatability disputable (*N* = 24)
*ALG14*, *BSCL2*, *BSCL2*, *CPT2*, *CYP11B1*, *EFL1*, *FLAD1*, *FTCD*, *GBE1*, *HADHB*, *HADHB*, *HNF4A*, *LBR*, *PARN*, *PKLR*, *SBDS*, *SDHA*, *SLC19A2*, *TBC1D24*, *TBK1*, *TRMT10A*, *TRMU*, *TSEN54*, *VAMP2*
7. Treatment options are promising but still experimental (*N* = 19)
*ALDH5A1*, *ALPL*, *AMPD1*, *APOA1*, *ATAD1*, *DHODH*, *GFPT1*, *GNS*, *GOT2*, *HGSNAT*, *MAN2B1*, *MOCS1*, *NAGLU*, *PDHB*, *PDXK*, *PIGA*, *SGSH*, *SLC6A8*, *TF*
8. No evidence that early detection and early treatment before clinical presentation leads to considerable benefit (*N* = 64)
*AASS*, *ABCA1*, *ABCB6*, *ABCB7*, *ALAS2*, *APOA1*, *CBLIF*, *CERS3*, *CYP11B1*, *FXN*, *G6PD*, *GALM*, *GATA1*, *GSTZ1*, *HK1*, *HOGA1*, *HPS1*, *HPS4*, *ITPR2*, *LACC1*, *LMAN1*, *MCFD2*, *MTHFR*, *MYO5A*, *NBEAL2*, *NR1H4*, *OAS1*, *PDX1*, *PFKM*, *PGK1*, *PMVK*, *PNPLA1*, *PPM1K*, *PRKCSH*, *PSTPIP1*, *RPL11*, *RPL15*, *RPL18*, *RPL26*, *RPL27*, *RPL35*, *RPL35A*, *RPL5*, *RPS10*, *RPS15A*, *RPS17*, *RPS19*, *RPS24*, *RPS26*, *RPS27*, *RPS28*, *RPS29*, *RPS7*, *SEC23A*, *SEC23B*, *SLC25A13*, *SLC52A1*, *SLC6A1*, *TRNT1*, *TSR2*, *TYMP*, *UNG*, *VMA21*, *VPS13D*
9. Positive treatment response in less than 75% of patients (*N* = 6)
*ADK*, *ARSA*, *COQ8A*, *MTR*, *MTRR*, *PDSS1*
10. Partially treatable disorders in which the available treatment has insufficient effect and/or critical manifestations of the disorder cannot be prevented (*N* = 43)
*ABCD4*, *ACSF3*, *AMT*, *ARSB*, *ATP13A2*, *CD320*, *CUBN*, *CYB561*, *DGKE*, *DHCR7*, *DHFR*, *DLAT*, *DLD*, *GATM*, *GCH1*, *GLDC*, *GLRX5*, *GRHPR*, *GUSB*, *HCFC1*, *HPD*, *IDS*, *MLYCD*, *MMACHC + PRDX1*, *MTHFS*, *NOLA2*, *NOLA3*, *PDHA1*, *PDHX*, *PDP1*, *PHGDH*, *PIGM*, *PIGO*, *PNP*, *PRPS1*, *PRPS1*, *SLC16A1*, *SLC25A32*, *SUCLA2*, *SUCLG1*, *TH*, *THAP11*, *ZNF143*
**Genes excluded for other reasons (*N* = 187)**
11. Causes disorders not within the field of IMD pediatricians in the Netherlands ** (*N* = 30)
*AKR1C2*, *AR*, *AR*, *CTPS1*, *CYP11A1*, *CYP19A1*, *CYP19A1*, *CYP21A2*, *ESR1*, *ESR2*, *GRM6*, *H6PD*, *HJV*, *HSD11B1*, *HSD11B2*, *HSD17B3*, *JAGN1*, *MAOA + MAOB*, *NR3C1*, *NR3C2*, *NR3C2*, *PGR*, *POR*, *RPL13*, *RPSA*, *SAR1B*, *SRD5A2*, *STEAP3*, *STS*, *SULT2B1*
12. Disorders considered to be benign, mild, or not clinically relevant for NBS (*N* = 39)
*AAGAB*, *ABCC2*, *AGXT2*, *AK7*, *ALB*, *ALDH4A1*, *AP2S1*, *CTH*, *CYCS*, *DCXR*, *HAL*, *HGD*, *KHK*, *LCT*, *LDHB*, *LIPH*, *LPAR6*, *MLPH*, *PCSK1*, *PHKA2*, *PHKB*, *PRODH2*, *PRRT2*, *PYGL*, *PYGM*, *RPL21*, *SARDH*, *SELENBP1*, *SLC16A1*, *SLC27A4*, *SLC30A2*, *SLC36A2*, *SLC36A2 (± SLC6A20)*, *SLC3A1*, *SLC7A9*, *SLCO1B1 + SLCO1B3*, *TDO2*, *TMEM199*, *TREH*
13. Clinical onset at age 10 years and older (*N* = 27)
*ABCC8*, *ABCC8*, *ABCG5*, *ABCG8*, *APOB*, *APOB*, *BLVRA*, *BMP6*, *CHCHD2*, *CPOX*, *CPT1C*, *DNAJC5*, *FDPS*, *GRN*, *HMBS*, *LDLR*, *LDLRAP1*, *MVD*, *NT5E*, *PCSK9*, *PHKA1*, *POFUT1*, *POLR3H*, *PPOX*, *SDHA*, *UROD*, *VPS13A*
14. Genes that also predispose for non-preventable and non-treatable disorders later in life (*N* = 22)
*APPL1*, *BLK*, *CYC1*, *DLST*, *DNAJC6*, *HNF1A*, *HNF1B*, *KIF5A*, *KLF11*, *LRRK2*, *MTAP*, *NEUROD1*, *NPM1*, *PAX4*, *PINK1*, *PRKN*, *SDHAF2*, *SDHB*, *SDHC*, *SDHD*, *UCP2*, *VPS13C*
15. Clinical diagnosis (at birth) or symptomatic *** (*N* = 26)
*ABCA12*, *ABCB11*, *ABCB4*, *ALAS2*, *ALOX12B*, *ALOXE3*, *ATP8B1*, *CHST11*, *CHST3*, *CTSB*, *CYP17A1*, *ENPP1*, *EOGT*, *EXT1*, *EXT2*, *FMO3*, *FTL*, *IMPAD1*, *PAPSS2*, *SDR9C7*, *SLC10A7*, *SLC26A2*, *TRAPPC2*, *TRIP11*, *UBIAD1*, *UROS*
16. Lack of consensus between the reviewers in the core team (*N* = 43)
*ACADS*, *AK2*, *AKT2*, *ALAD*, *APOA5*, *APRT*, *COQ2*, *COQ5*, *COQ6*, *COQ7*, *COQ9*, *CP*, *CTNS*, *EPHX1*, *GALE*, *GGCX*, *HAMP*, *HFE2*, *HSD3B2*, *LDHA*, *LMBRD1*, *LMF1*, *MC2R*, *MMADHC*, *MMADHC*, *MRAP*, *PDSS2*, *PHKG2*, *PMM2*, *PPA2*, *SLC30A10*, *SLC35A2*, *SLC39A14*, *SLC39A4*, *SLC39A8*, *SLC5A6*, *SLC7A7*, *SPR*, *STAR*, *TFR2*, *TMLHE*, *UMPS*, *VKORC1*

A list of the names of the inherited metabolic disorders and corresponding OMIM codes can be requested from the authors. ** Some IMDs in the list of 1459 disorders are, in principle, an IMD, but they are not a focus within the expertise of the Dutch pediatricians treating IMDs in the Netherlands. *** These include, e.g., IMDs that have a dermatological presentation at birth or a distinctive symptomatic phenotype in which diagnostics are preferred over screening. IMDs = Inherited Metabolic Disorders, NBS = Newborn Screening.

## Appendix C

**Table A3:** Reasons for IMD inclusion or exclusion by the core and project teams.

IMD	Associated Gene(s)	MIM	Argument(s) to Exclude Gene(s) by the Core Team During the Literature Review	Reason(s) for Final Decision to (Re)Include Gene(s) by the Project Team After Meeting 2
1. AKT2 superactivity (synonym: hypoinsulinemic hypoglycemia with hemihypertrophy)	*AKT2*	*164731, #240900	Discussion within the core team—several patients died from hypoglycemia within hours, so rapid diagnosis could be life-saving. However, results cannot be provided this rapidly. In addition, there was not enough evidence in the literature for other treatments.	*AKT2* was added because the project team decided that the benefit of preventing hypoglycemic episodes, and therefore indirectly preventing complications in newborns, leads to considerable health benefits. Hepatic glycogen synthase deficiency (*GYS2*, MIM *138571, #240600) had already been included based on this reasoning.
2. Arylsulfatase A deficiency	*ARSA*	*607574, #250100	Enzyme therapy does not improve outcome in every patient, according to Kaminski et al. [66]. Gene therapy is in development in mice. The core team concluded there was not enough evidence for effective treatment in >75% of patients.	Treatability highly debatable, but gene therapy was approved by the European Medicines Agency in 2020.
3. Carnitine palmitoyltransferase 2 deficiency	*CPT2*	*600650 #614212, #600649, #608836, #255110	First, there are major uncertainties in the natural course of these patients. Second, the Dutch Health Council had advised against the inclusion of CPT2 because of a large phenotypic variation in the severity of the disorder and the risk of overtreatment [34].	Already a candidate for expansion of NBS. *
4. Carnitine-acylcarnitine translocase deficiency	*SLC25A20*	*613698, #212138	Poor prognosis, with most patients dying within 3 months, and only a few who were treated early had a favorable outcome in the medium term [ORPHA: 159].	Already a candidate for expansion of NBS. *
5. CTP synthase 1 deficiency	*CTPS1*	*123860 #615897	IMD is not treated within the IMD departments in the Netherlands.	IMD is treatable by hemopoietic stem cell transplant. While invasive, this is crucial to survive infections early in life.
6. Cystinosis	*CTNS*	*606272 #219800, #219900, #219750	Highly disputed within the core team. Excluded because one of the four phenotypes has an adult-onset.	The benefit of starting early in newborns is clear according to Hohenfellner et al. [67] (except for renal Fanconi syndrome).
7. Hemojuvelin deficiency	*HJV*	*608374 *#602390*	IMD is not treated within the IMD departments in the Netherlands.	Early detection of the disorder is important because iron depletion by phlebotomy can prevent organ damage and all disease manifestations, see Roetto et al. [68].
8. Purine nucleoside phosphorylase deficiency	*PNP*	*164050 #613179	Not treatable according to van Karnebeek et al. [46,47]. Highly invasive hemopoietic stem cell transplant is crucial to survive infections early in life.	The efficacy of treatment depends on an early approach, see La Marca et al. [69].
9. Tyrosine hydroxylase deficiency	*TH*	*191290 #605407	A progressive, often lethal, neurometabolic disorder that can be improved but not cured by L-dopa, see Hoffman et al. [70] and de Lonlay et al. [71].	Only a very few patients respond poorly (or not at all). These patients have two severe mutations. According to Willemsen et al. [72], early diagnosis and treatment improve final outcome with regard to motor and cognitive functions.
			**Argument(s) to Include Gene(s) by the Core Team During the Literature Review**	**Reason(s) for Final Decision to Exclude Gene(s) by the Project Team After Meeting 2**
10. Mitochondrial thiamine pyrophosphate transporter deficiency	*SLC25A19*	*606521 #613710 #607196	With early diagnosis and immediate start of optimal treatment, the prognosis improves in most patients.	Excluded due to the existence of a lethal variant (Marcé-Grau et al. [73]), according to criterion 6 in Figure 1: Variable phenotype, with the treatability of one phenotype disputable.

* See Discussion Section 4.3. In June 2024 the Dutch Health Council declined to include mitochondrial acetoacetyl-CoA thiolase deficiency (*ACAT1*, MIM #203750), carnitine palmitoyl-transferase 2 deficiency (*CPT2*, MIM #614212, #600649, #608836, #255110), and carnitine-acylcarnitine translocase deficiency (*SLC25A20*, MIM #212138) given uncertainties in the natural course of these disorders.

## Appendix D. Discussion on IMDs from the Current NBS and the Secondary Findings

The genes related to disorders in current NBS elicited discussion. Trifunctional protein deficiency/long-chain hydroxyacyl-CoA dehydrogenase deficiency (*HADHA*, MIM *600890, #609016, #609015; *HADHB*, MIM *143450, #620300) revealed a flaw in the approach of automatically placing the biomarker-NBS IMDs into the final list. The *HADHA* and *HADHB* genes cannot be discriminated biochemically, but they can be differentiated using NGS, with *HADHA* regarded as treatable in the context of NBS, whereas *HADHB* is not due to its extreme phenotypic variability [74]. Novel mutations in *HADHB* cause a mild phenotype of mitochondrial trifunctional protein (MTP) deficiency [62]. With this knowledge, both the core and project teams could not justify including *HADHB* in the final list. We also removed MMA type cblB (*MMAB*, MIM *607568, #251110), which is one of the targeted genes causing an increase in the biomarker methylmalonic acid from the Dutch governmental advisory report [13]. This gene was removed because it has insufficient published evidence for the effectiveness of treatment.

A second challenge was to what extent the genes that are incidental findings in the current NBS should be included in our final list. These are the secondary findings due to 15 IMDs and their associated genes (*N* = 32) in Table 1. The decision to include these genes was based on the fact that patients are already detected by the current NBS. In particular, these are genes found by, e.g., hyperphenylalaninemia or by abnormal acylcarnitine concentrations. Keeping our criteria from Figure 1 in mind, the treatability of many of these secondary findings is disputable. This led us to take a closer and more critical look at the secondary finding. For methylmalonic acidemia (alone or in combination with homocystinuria), the list of associated genes is extensive. The governmental guidance in the Netherlands is rather unclear on the precise definition of MMA for inclusion in NBS, targeted genes as presumed from the advisory report include *MMUT*, MIM *609058, #251000; *MMAA*, MIM *607481, #251100; *MMAB*, MIM *607568, #251110; *MMADHC*, MIM *609831, #277410; *MMACHC*, MIM *611935, #277400; and *MCEE*, MIM *608419 #251120) [13]. In addition, the treatability of a large number of these phenotypes was questionable, and we therefore excluded the following genes: *LMBRD1* (MIM *612625, #277380) *SUCLA2* (MIM *603921, #612073), *SUCLG1* (MIM *611224, #245400), *MLYCD* (MIM *606761, #248360), *ACSF3* (MIM *614245, #614265), *PRDX1* (MIM *176763, #277400), *ABCD4* (MIM *603214, #614857), *HCFC1* (MIM *300019, #309541), *THAP11* (MIM *609119), *CD320* (MIM *606475, #613646), *CBLIF* (MIM *609342, #261000), *CUBN* (MIM *602997, #261100, #618884), and *ZNF143* (MIM *603433).

Furthermore, maleylacetoacetate isomerase deficiency (*GSTZ1*, MIM *603758, #617596) and riboflavin transporter 1 deficiency (synonym: transient riboflavin deficiency) (*SLC52A1*, MIM *607883, #615026) were excluded because they have a very mild or benign phenotype. In addition, 2-methyl-3-hydroxybutyryl-CoA dehydrogenase deficiency (HSD10 disease) (*HSD17B10*, MIM *300256, #300438) was excluded because the prognosis is unacceptably poor for NBS, 3-methylglutaconyl-CoA hydratase deficiency (*AUH*, MIM *600529, #250950) was excluded because there is insufficient evidence of effective treatment and the natural course of the disorders is largely unknown, and flavin adenine dinucleotide synthetase deficiency (*FLAD1*, MIM *610595, #255100) was excluded because of its extreme phenotypic variability, in which the severe variant is invariably fatal.

## Appendix E. Considerations for the Final List of Genes, Part 2

The project team also opted to reconsider the exclusion of the Vitamin B6-dependent epilepsies, such as pyridoxamine 5′-phosphate oxidase deficiency (*PNPO*, MIM *603287, #610090) and pyridoxal 5′-phosphate binding protein deficiency (*PROSC*, MIM *604436, #617290), from the 100 genes. However, excluding these IMDs was felt to be inconsistent because we had included a similar IMD, α-aminoadipic semialdehyde dehydrogenase deficiency (*ALDH7A1*, MIM *107323 #266100), with no debate.

Other inherited disorders of carbohydrate metabolism for which the exclusion could be reconsidered according to the project team were: glycogen branching enzyme deficiency (*GBE1*, MIM *607839, #232500, #263570), hepatic phosphorylase kinase α2 subunit deficiency (*PHKA2*, MIM *300798, #306000), phosphorylase kinase β subunit deficiency *(PHKB*, MIM *172490, #261750), hepatic phosphorylase kinase γ2 subunit deficiency (*PHKG2*, MIM *172471 #613027), hepatic glycogen phosphorylase deficiency (*PYGL*, MIM *613741, #232700), and intestinal sodium-glucose cotransporter 1 deficiency (*SLC5A1*, MIM *182380, #606824). However, these remain excluded for now for the reasons detailed in Appendix B.

Members of the project team did highlight some other IMDs for further in-depth discussion. These included 5,10-methylenetetrahydrofolate reductase deficiency (*MTHFR*, MIM *607093 #236250), biotin-responsive basal ganglia disease (*SLC19A3*, MIM *606152 #607483), Molybdenum cofactor deficiency type A (*MOCS1*, MIM *603707 #252150), and TANGO2 deficiency (*TANGO2*, MIM *616830, #616878).

## References

[1] TNO (2021). The Newborn Blood Spot Screening Monitor 2019.

[2] TNO (2022). The Newborn Blood Spot Screening Monitor 2020.

[3] TNO (2023). The Newborn Blood Spot Screening Monitor 2021.

[4] TNO (2023). The Newborn Blood Spot Screening Monitor 2022.

[5] (2024). National Institute for Public Health and Environment. Pre- and Neonatal Screenings (PNS), Heel Prick Screening Test [1]. Ministry of Health, Welfare and Sport. https://www.pns.nl/en/prenatal-and-newborn-screening/heel-prick-screening-test.

[6] (2024). National Institute for Public Health and Environment. Pre- and Neonatal Screenings (PNS), Clinical Picture. Ministry of Health, Welfare and Sport. https://www.pns.nl/en/heel-prick/clinical-picture.

[7] van Spronsen F.J., Blau N., Harding C., Burlina A., Longo N., Bosch A.M. (2021). Phenylketonuria. Nat. Rev. Dis. Primers.

[8] van Vliet K., Dijkstra A.M., Bouva M.J., van der Krogt J., Bijsterveld K., van der Sluijs F., Velden M.G.d.S.d., Koop K., Rossi A., Thomas J.A. (2023). Maleic acid is a biomarker for maleylacetoacetate isomerase deficiency; implications for newborn screening of tyrosinemia type 1. J. Inherit. Metab. Dis..

[9] Yang H., Al-Hertani W., Cyr D., Laframboise R., Parizeault G., Wang S.P., Rossignol F., Berthier M.-T., Giguère Y., Waters P.J. (2017). Hypersuccinylacetonaemia and normal liver function in maleylacetoacetate isomerase deficiency. J. Med. Genet..

[10] Hagemeijer M.C., Oussoren E., Ruijter G.J.G., Onkenhout W., Huidekoper H.H., Ebberink M.S., Waterham H.R., Ferdinandusse S., de Vries M.C., Huigen M.C.D.G. (2021). Abnormal VLCADD newborn screening resembling MADD in four neonates with decreased riboflavin levels and VLCAD activity. JIMD Rep..

[11] Lee S.H., Hong Y.H. (2014). Asymptomatic maternal 3-methylcrotonylglycinuria detected by her unaffected baby’s neonatal screening test. Korean J. Pediatr..

[12] (2024). Health Council of the Netherlands. Neonatale Hielprikscreening op Severe Combined Immunodeficiency (SCID). https://www.gezondheidsraad.nl/onderwerpen/preventie/alle-adviezen-over-preventie/neonatale-hielprikscreening-op-severe-combined-immunodeficiency-scid#:~:text=In%202021%20is%20SCID%20toegevoegd,SCID%20onder%20de%20doelziekte%20geschaard.

[13] Health Council of the Netherlands (2005). Neonatal Screening.

[14] Adhikari A.N., Gallagher R.C., Wang Y., Currier R.J., Amatuni G., Bassaganyas L., Chen F., Kundu K., Kvale M., Mooney S.D. (2020). The role of exome sequencing in newborn screening for inborn errors of metabolism. Nat. Med..

[15] Boemer F., Fasquelle C., D’otreppe S., Josse C., Dideberg V., Segers K., Guissard V., Capraro V., Debray F., Bours V. (2017). A next-generation newborn screening pilot study: NGS on dried blood spots detects causal mutations in patients with inherited metabolic diseases. Sci. Rep..

[16] Kingsmore S.F., Smith L.D., Kunard C.M., Bainbridge M., Batalov S., Benson W., Blincow E., Caylor S., Chambers C., Del Angel G. (2022). A genome sequencing system for universal newborn screening, diagnosis, and precision medicine for severe genetic diseases. Am. J. Hum. Genet..

[17] Veldman A., Kiewiet M.B.G., Heiner-Fokkema M.R., Nelen M.R., Sinke R.J., Sikkema-Raddatz B., Voorhoeve E., Westra D., Dollé M.E.T., Schielen P.C.J.I. (2022). Towards Next-Generation Sequencing (NGS)-Based Newborn Screening: A Technical Study to Prepare for the Challenges Ahead. Int. J. Neonatal Screen..

[18] Strand J., Gul K.A., Erichsen H.C., Lundman E., Berge M.C., Trømborg A.K., Sørgjerd L.K., Ytre-Arne M., Hogner S., Halsne R. (2020). Second-Tier Next Generation Sequencing Inte-grated in Nationwide Newborn Screening Provides Rapid Molecular Diagnostics of Severe Combined Immunodeficiency. Front. Immunol..

[19] Chan T.C.H., Mak C.M., Yeung M.C.W., Law E.C.-Y., Cheung J., Wong T.K., Cheng V.W.-S., Lee J.K.H., Wong J.C.L., Fung C.W. (2024). Harnessing Next-Generation Sequencing as a Timely and Accurate Second-Tier Screening Test for Newborn Screening of Inborn Errors of Metabolism. Int. J. Neonatal Screen..

[20] Trier C., Fournous G., Strand J.M., Stray-Pedersen A., Pettersen R.D., Rowe A.D. (2020). Next-generation sequencing of newborn screening genes: The accuracy of short-read mapping. npj Genom. Med..

[21] van Campen J.C., Sollars E.S., Thomas R.C., Bartlett C.M., Milano A., Parker M.D., Dawe J., Winship P.R., Peck G., Grafham D. (2019). Next Generation Sequencing in Newborn Screening in the United Kingdom National Health Service. Int. J. Neonatal Screen..

[22] Holm I.A., Agrawal P.B., Ceyhan-Birsoy O., Christensen K.D., Fayer S., Frankel L.A., Genetti C.A., Krier J.B., LaMay R.C., Levy H.L. (2018). The BabySeq project: Implementing genomic sequencing in newborns. BMC Pediatr..

[23] Yang R.L., Qian G.L., Wu D.W., Miao J.K., Yang X., Wu B.Q., Yan Y.Q., Li H.B., Mao X.M., He J. (2023). A multicenter prospective study of next-generation sequenc-ing-based newborn screening for monogenic genetic diseases in China. World J. Pediatr..

[24] La Marca G., Carling R.S., Moat S.J., Yahyaoui R., Ranieri E., Bonham J.R., Schielen P.C.J.I. (2023). Current State and Innovations in Newborn Screening: Continuing to Do Good and Avoid Harm. Int. J. Neonatal Screen..

[25] Stenton S.L., Campagna M., Philippakis A., O’Donnell-Luria A., Gelb M.H. (2023). First-tier next-generation sequencing for newborn screening: An important role for biochemical second-tier testing. Genet. Med. Open.

[26] Kiewiet G., Westra D., de Boer E.N., van Berkel E., Hofste T.G., van Zweeden M., Derks R.C., Leijsten N.F., Ruiterkamp-Versteeg M.H., Charbon B. (2024). Future of Dutch NGS-Based Newborn Screening: Exploring the Technical Possibilities and Assessment of a Variant Classification Strategy. Int. J. Neonatal Screen..

[27] Chen T., Fan C., Huang Y., Feng J., Zhang Y., Miao J., Wang X., Li Y., Huang C., Jin W. (2023). Genomic Sequencing as a First-Tier Screening Test and Outcomes of Newborn Screening. JAMA Netw. Open.

[28] Jiang S., Wang H., Gu Y. (2023). Genome Sequencing for Newborn Screening—An Effective Approach for Tackling Rare Diseases. JAMA Netw. Open.

[29] Spiekerkoetter U., Bick D., Scott R., Hopkins H., Krones T., Gross E.S., Bonham J.R. (2023). Genomic newborn screening: Are we entering a new era of screening?. J. Inherit. Metab. Dis..

[30] Green R.C., Shah N., Genetti C.A., Yu T., Zettler B., Uveges M.K., Ceyhan-Birsoy O., Lebo M.S., Pereira S., Agrawal P.B. (2023). Actionability of unanticipated monogenic disease risks in newborn genomic screening: Findings from the BabySeq Project. Am. J. Hum. Genet..

[31] Andermann A., Blancquaert I., Beauchamp S., Dery V. (2008). Revisiting Wilson and Jungner in the genomic age: A review of screening criteria over the past 40 years. Bull World Health Organ.

[32] Wilson J.M.G., Jungner G., World Health Organization, World Health Organization (1968). Principles and Practice of Screening for Disease.

[33] Sturdy S., Miller F., Hogarth S., Armstrong N., Chakraborty P., Cressman C., Dobrow M., Flitcroft K., Grossman D., Harris R. (2020). Half a Century of Wilson & Jungner: Reflections on the Governance of Population Screening. Wellcome Open Res..

[34] Gezondheidsraad, Ministerie van Volksgezond-Heid WeS (2020). Advies Screenen op Niet-Behandelbare Aandoeningen Vroeg in het Leven.

[35] Lombardo S., Seedat F., Elliman D., Marshall J. (2023). Policy-making and implementation for newborn bloodspot screening in Europe: A comparison between EURORDIS principles and UK practice. Lancet Reg. Health–Eur..

[36] Kalkman S., Dondorp W. (2022). The case for screening in early life for ‘non-treatable’ disorders: Ethics, evidence and proportionality. A report from the Health Council of the Netherlands. Eur. J. Hum. Genet..

[37] Bick D., Ahmed A., Deen D., Ferlini A., Garnier N., Kasperaviciute D., Leblond M., Pichini A., Rendon A., Satija A. (2022). Newborn Screening by Genomic Sequencing: Opportunities and Challenges. Int. J. Neonatal Screen..

[38] Jansen M.E., Klein A.W., Buitenhuis E.C., Rodenburg W., Cornel M.C. (2021). Expanded Neonatal Bloodspot Screening Programmes: An Evaluation Framework to Discuss New Conditions With Stakeholders. Front. Pediatr..

[39] Veldman A., Kiewiet M.B.G., Westra D., Bosch A.M., Brands M.M.G., de Coo R.I.F.M., Derks T.G.J., Fuchs S.A., Hout J.M.P.v.D., Huidekoper H.H. (2023). A Delphi Survey Study to Formulate Statements on the Treatability of Inherited Metabolic Disorders to Decide on Eligibility for Newborn Screening. Int. J. Neonatal Screen..

[40] Watson M.S., Mann M.Y., Lloyd-Puryear M.A., Rinaldo P., Howell R.R. (2006). Newborn Screening: Toward a Uniform Screening Panel and System—Executive Summary. Pediatrics.

[41] Kemper A.R., Green N.S., Calonge N., Lam W.K., Comeau A.M., Goldenberg A.J., Ojodu J., Prosser L.A., Tanksley S., Bocchini J.A. (2014). Decision-making process for conditions nominated to the Recommended Uniform Screening Panel: Statement of the US Department of Health and Human Services Secretary’s Advisory Committee on Heritable Disorders in Newborns and Children. Genet. Med..

[42] Milko L.V., O’Daniel J.M., DeCristo D.M., Crowley S.B., Foreman A.K.M., Wallace K.E., Mollison L.F., Strande N.T., Girnary Z.S., Boshe L.J. (2019). An Age-Based Framework for Evaluating Genome-Scale Sequencing Results in Newborn Screening. J. Pediatr..

[43] Children ACoHDiNa, (HHS) DoHaHS (2018). Recommended Uniform Screening Panel.

[44] (2018). Clinical Principal Committee, Standing Committee on Screening. Population-Based Screening Framework.

[45] Australian Health Ministers’ Advisory Council, 2018 DoH (2018). Newborn Bloodspot Screening National Policy Framework.

[46] Van Karnebeek C.D.M., Shevell M., Zschocke J., Moeschler J.B., Stockler S. (2014). The metabolic evaluation of the child with an intellectual developmental disorder: Diagnostic algorithm for identification of treatable causes and new digital resource. Mol. Genet. Metab..

[47] van Konijnenburg E.M.M.H., Wortmann S.B., Koelewijn M.J., Tseng L.A., Houben R., Stöckler-Ipsiroglu S., Ferreira C.R., van Karnebeek C.D.M. (2021). Treatable inherited metabolic disorders causing intellectual disability: 2021 review and digital app. Orphanet J. Rare Diseases..

[48] Ferreira C.R., Rahman S., Keller M., Zschocke J., ICIMD Advisory Group (2021). An international classification of inherited metabolic disorders (ICIMD). J. Inherit. Metab. Dis..

[49] Ferreira C.R., Van Karnebeek C.D.M., Vockley J., Blau N. (2019). A proposed nosology of inborn errors of metabolism. Genet. Med..

[50] (2020). VSOP Patient Alliance for Rare and Genetic Diseases. Letter to the State Secretary in Response to the Health Council’s Report ‘Screening for Non-Treatable Disorders Early in Life’. https://vsopnl/actueel/de-vsop-pleit-voor-een-aangepast-besliskader-voor-de-hielprik/.

[51] van Dijk T., Kater A., Jansen M., Dondorp W.J., Blom M., Kemp S., Langeveld M., Cornel M.C., van der Pal S.M., Henneman L. (2021). Expanding Neonatal Bloodspot Screening: A Multi-Stakeholder Perspective. Front. Pediatr..

[52] Plass A.M.C., van El C.G., Pieters T., Cornel M.C. (2010). Neonatal Screening for Treatable and Untreatable Disorders: Prospective Parents’ Opinions. Pediatrics.

[53] (2021). EURORDIS—Rare Diseases Europe. Key Principles for Newborn Screening Eurordis.org/ Newbornscreening. https://www.eurordis.org/publications/key-principles-for-newborn-screening/.

[54] Zhou L., Deng J., Stenton S.L., Zhou J., Li H., Chen C., Prokisch H., Fang F. (2020). Case Report: Rapid Treatment of Uridine-Responsive Epileptic En-cephalopathy Caused by CAD Deficiency. Front. Pharmacol..

[55] Jager E.A., Kuijpers M.M., Bosch A.M., Mulder M.F., Gozalbo E.R., Visser G., de Vries M., Williams M., Waterham H.R., van Spronsen F.J. (2019). A nationwide retrospective observational study of population newborn screening for medium-chain acyl-CoA dehydrogenase (MCAD) deficiency in the Netherlands. J. Inherit. Metab. Dis..

[56] Jennions E., Hedberg-Oldfors C., Berglund A., Kollberg G., Törnhage C., Eklund E.A., Oldfors A., Verloo P., Vanlander A.V., De Meirleir L. (2019). TANGO2 deficiency as a cause of neurodevelopmental delay with indirect effects on mitochondrial energy metabolism. J. Inherit. Metab. Dis..

[57] Cavicchi C., Oussalah A., Falliano S., Ferri L., Gozzini A., Gasperini S., Motta S., Rigoldi M., Parenti G., Tummolo A. (2021). *PRDX1* gene-related *epi-cblC* disease is a common type of inborn error of cobalamin metabolism with mono- or bi-allelic MMACHC epimutations. Clin. Epigenet..

[58] Guéant J.-L., Chéry C., Oussalah A., Nadaf J., Coelho D., Josse T., Flayac J., Robert A., Koscinski I., Gastin I. (2018). A PRDX1 mutant allele causes a MMACHC secondary epimutation in cblC patients. Nat. Commun..

[59] Gold N.B., Adelson S.M., Shah N., Williams S., Bick S.L., Zoltick E.S., Gold J.I., Strong A., Ganetzky R., Roberts A.E. (2023). Perspectives of Rare Disease Experts on Newborn Genome Sequencing. JAMA Netw. Open.

[60] Minten T., Gold N.B., Bick S., Adelson S., Gehlenborg N., Amendola L.M., Boemer F., Coffey A.J., Encina N., Ferlini A. (2024). Data-driven prioritization of genetic disorders for global genomic newborn screening programs. medRxiv.

[61] Ziegler A., Koval-Burt C., Kay D.M., Suchy S.F., Begtrup A., Langley K.G., Hernan R., Amendola L.M., Boyd B.M., Bradley J. (2024). Expanded Newborn Screening Using Genome Sequencing for Early Actionable Conditions. JAMA.

[62] Milko L.V., Berg J.S. (2023). Age-Based Genomic Screening during Childhood: Ethical and Practical Considerations in Public Health Genomics Implementation. Int. J. Neonatal Screen..

[63] Burlina A., Jones S.A., Chakrapani A., Church H.J., Heales S., Wu T.H.Y., Morton G., Roberts P., Sluys E.F., Cheillan D. (2022). A New Approach to Objectively Evaluate Inherited Metabolic Diseases for Inclusion on Newborn Screening Programmes. Int. J. Neonatal Screen..

[64] Kelly N., Makarem D.C., Wasserstein M.P. (2016). Screening of Newborns for Disorders with High Benefit-Risk Ratios Should Be Mandatory. J. Law Med. Ethics.

[65] Zaidman C.M., Crockett C.D., Wedge E., Tabatabai G., Goedeker N. (2024). Newborn Screening for Spinal Muscular Atrophy: Variations in Practice and Early Management of Infants with Spinal Muscular Atrophy in the United States. Int. J. Neonatal Screen..

[66] Kaminski D., Yaghootfam C., Matthes F., Reßing A., Gieselmann V., Matzner U. (2021). Brain cell type-specific endocytosis of arylsulfatase A identifies limitations of enzyme-based therapies for metachromatic leukodystrophy. Hum. Mol. Genet..

[67] Hohenfellner K., Rauch F., Ariceta G., Awan A., Bacchetta J., Bergmann C., Bechtold S., Cassidy N., Deschenes G., Elenberg E. (2019). Management of bone disease in cystinosis: Statement from an international conference. J. Inherit. Metab. Dis..

[68] Roetto A., Totaro A., Cazzola M., Cicilano M., Bosio S., D’Ascola G., Carella M., Zelante L., Kelly A., Cox T. (1999). Juvenile Hemochromatosis Locus Maps to Chromosome 1q. Am. J. Hum. Genet..

[69] La Marca G., Canessa C., Giocaliere E., Romano F., Malvagia S., Funghini S., Moriondo M., Valleriani C., Lippi F., Ombrone D. (2014). Diagnosis of immunodeficiency caused by a purine nucleoside phosphorylase defect by using tandem mass spectrometry on dried blood spots. J. Allergy Clin. Immunol..

[70] Hoffmann G.F., Assmann B., Dionisi-Vici C., De Klerk J.B.C., Naumann M., Steenbergen-Spanjers G.C.H., Strassburg H.-M., Wevers R.A., Bräutigam C., Häussler M. (2003). Tyrosine hydroxylase deficiency causes progressive encephalopathy and dopa-nonresponsive dystonia. Ann. Neurol..

[71] De Lonlay P., Nassogne M.C., van Gennip A.H., van Cruchten A.C., de Villemeur T.B., Cretz M., Stoll C., Launay J.M., Steenberger-Spante G.C.V., Heuvel L.P.W.v.D. (2000). Tyrosine hydroxylase deficiency unresponsive to L-dopa treatment with unusual clinical and biochemical presentation. J. Inherit. Metab. Dis..

[72] Willemsen M.A., Verbeek M.M., Kamsteeg E.J., de Rijk-van Andel J.F., Aeby A., Blau N., Burlina A., Donati M.A., Geurtz B., Grattan-Smith P.J. (2010). Tyrosine hydroxylase deficiency: A treatable disorder of brain catecholamine biosynthesis. Brain.

[73] Marcé-Grau A., Martí-Sánchez L., Baide-Mairena H., Ortigoza-Escobar J.D., Pérez-Dueñas B. (2019). Genetic defects of thiamine transport and metabolism: A review of clinical phenotypes, genetics, and functional studies. J. Inherit. Metab. Dis..

[74] Ørstavik K., Arntzen K.A., Mathisen P., Backe P.H., Tangeraas T., Rasmussen M., Kristensen E., Van Ghelue M., Jonsrud C., Bliksrud Y.T. (2022). Novel mutations in the HADHB gene causing a mild phenotype of mitochondrial trifunctional protein MTP deficiency. JIMD Rep..

